# RNA-Seq workflow: gene-level exploratory analysis and differential expression

**DOI:** 10.12688/f1000research.7035.1

**Published:** 2015-10-14

**Authors:** Michael I. Love, Simon Anders, Vladislav Kim, Wolfgang Huber

**Affiliations:** 1 Department of Biostatistics and Computational Biology, Dana-Farber Cancer Institute and Department of Biostatistics, Harvard TH Chan School of Public Health, Boston, Massachusetts, USA; 2 Institute for Molecular Medicine Finland, Helsinki, Finland; 3 European Molecular Biology Laboratory, Heidelberg, Germany

**Keywords:** RNA-seq, differential expression, gene expression, Bioconductor, statistical analysis, high-throughput sequencing, visualization, genomics

## Abstract

Here we walk through an end-to-end gene-level RNA-Seq differential expression workflow using Bioconductor packages. We will start from the FASTQ files, show how these were aligned to the reference genome, and prepare a count matrix which tallies the number of RNA-seq reads/fragments within each gene for each sample. We will perform exploratory data analysis (EDA) for quality assessment and to explore the relationship between samples, perform differential gene expression analysis, and visually explore the results.

## Introduction

Bioconductor has many packages which support analysis of high-throughput sequence data, including RNA sequencing (RNA-seq). The packages which we will use in this workflow include core packages maintained by the Bioconductor core team for importing and processing raw sequencing data and loading gene annotations. We will also use contributed packages for statistical analysis and visualization of sequencing data. Through scheduled releases every 6 months, the Bioconductor project ensures that all the packages within a release will work together in harmony (hence the “conductor” metaphor). The packages used in this workflow are loaded with the *library* function and can be installed by following the Bioconductor package installation instructions.

If you have questions about this workflow or any Bioconductor software, please post these to the Bioconductor support site. If the questions concern a specific package, you can tag the post with the name of the package, or for general questions about the workflow, tag the post with rnaseqgene. Note the posting guide for crafting an optimal question for the support site.

### Experimental data

The data used in this workflow is stored in the *airway* package that summarizes an RNA-seq experiment wherein airway smooth muscle cells were treated with dexamethasone, a synthetic glucocorticoid steroid with anti-inflammatory effects ^1^. Glucocorticoids are used, for example, by people with asthma to reduce inflammation of the airways. In the experiment, four primary human airway smooth muscle cell lines were treated with 1 micromolar dexamethasone for 18 hours. For each of the four cell lines, we have a treated and an untreated sample. For more description of the experiment see the PubMed entry 24926665 and for raw data see the GEO entry GSE52778.

## Preparing count matrices

As input, the count-based statistical methods, such as *DESeq2* ^2^, *edgeR* ^3^, *limma* with the voom method ^4^, *DSS* ^5^, *EBSeq* ^6^ and *BaySeq* ^7^, expect input data as obtained, e.g., from RNA-seq or another high-throughput sequencing experiment, in the form of a matrix of integer values. The value in the *i*-th row and the *j*-th column of the matrix tells how many reads (or fragments, for paired-end RNA-seq) have been unambiguously assigned to gene *i* in sample *j*. Analogously, for other types of assays, the rows of the matrix might correspond e.g., to binding regions (with ChIP-Seq), species of bacteria (with metagenomic datasets), or peptide sequences (with quantitative mass spectrometry).

The values in the matrix must be raw counts of sequencing reads/fragments. This is important for *DESeq2*’s statistical model to hold, as only the raw counts allow assessing the measurement precision correctly. It is important to *never* provide counts that were pre-normalized for sequencing depth/library size, as the statistical model is most powerful when applied to raw counts, and is designed to account for library size differences internally.

### Aligning reads to a reference genome

The computational analysis of an RNA-seq experiment begins earlier: we first obtain a set of FASTQ files that contain the nucleotide sequence of each read and a quality score at each position. These reads must first be aligned to a reference genome or transcriptome. It is important to know if the sequencing experiment was single-end or paired-end, as the alignment software will require the user to specify both FASTQ files for a paired-end experiment. The output of this alignment step is commonly stored in a file format called SAM/BAM.

A number of software programs exist to align reads to a reference genome, and the development is too rapid for this document to provide an up-to-date list. We recommend consulting benchmarking papers that discuss the advantages and disadvantages of each software, which include accuracy, sensitivity in aligning reads over splice junctions, speed, memory footprint, usability, and many other features.

The reads for this experiment were aligned to the Ensembl release 75 ^8^ human reference genome using the STAR read aligner ^9^. In this example, we have a file in the current directory called files with each line containing an identifier for each experiment, and we have all the FASTQ files in a subdirectory fastq. If you have downloaded the FASTQ files from the Sequence Read Archive, the identifiers would be SRA run IDs, e.g. SRR1039520. You should have two files for a paired-end experiment for each ID, fastq/SRR1039520_1.fastq1 and fastq/SRR1039520_2.fastq, which give the first and second read for the paired-end fragments. If you have performed a single-end experiment, you would only have one file per ID. We have also created a subdirectory, aligned, where STAR will output its alignment files.



                        for f in 'cat files'; do STAR --genomeDir ../STAR/ENSEMBL.homo_sapiens.release-75 \
--readFilesIn fastq/$f\_1.fastq fastq/$f\_2.fastq \
--runThreadN 12 --outFileNamePrefix aligned/$f.; done
                    


SAMtools ^10^ was used to generate BAM files. The –@ flag can be used to allocate additional threads.



                        for f in 'cat files'; do samtools view -bS aligned/$f.Aligned.out.sam \
-o aligned/$f.bam; done
                    


The BAM files for a number of sequencing runs can then be used to generate count matrices, as described in the following section.

### Locating alignment files

Besides the count matrix that we will use later, the *airway* package also contains eight files with a small subset of the total number of reads in the experiment. The reads were selected which aligned to a small region of chromosome 1. We chose a subset of reads because the full alignment files are large (a few gigabytes each), and because it takes between 10–30 minutes to count the fragments for each sample. We will use these files to demonstrate how a count matrix can be constructed from BAM files. Afterwards, we will load the full count matrix corresponding to all samples and all data, which is already provided in the same package, and will continue the analysis with that full matrix.

We load the data package with the example data:



                        library
                        (
                        "airway"
                        )
                    


The R function *system.file* can be used to find out where on your computer the files from a package have been installed. Here we ask for the full path to the extdata directory, where R packages store external data, that is part of the *airway* package.



                        dir <- 
                        system.file
                        (
                        "extdata"
                        , 
                        package=
                        “airway"
                        , 
                        mustWork=
                        TRUE
                        )
                    


In this directory, we find the eight BAM files (and some other files):



                        list.files
                        (dir)
                    




                        ##  [1] "GSE52778_series_matrix.txt"	“Homo_sapiens.GRCh37.75_subset.gtf"
##  [3] "sample_table.csv"		"SraRunInfo_SRP033351.csv"
##  [5] "SRR1039508_subset.bam"		"SRR1039508_subset.bam.bai"
##  [7] "SRR1039509_subset.bam"		"SRR1039512_subset.bam"
##  [9] "SRR1039513_subset.bam"		"SRR1039516_subset.bam"
## [11] "SRR1039517_subset.bam"		"SRR1039520_subset.bam"
## [13] "SRR1039521_subset.bam"
                    


Typically, we have a table with detailed information for each of our samples that links samples to the associated FASTQ and BAM files. For your own project, you might create such a comma-separated value (CSV) file using a text editor or spreadsheet software such as Excel.

We load such a CSV file with *read.csv*:



                        csvfile <- 
                        file.path
                        (dir,
                        “sample_table.csv"
                        )
(sampleTable <- 
                        read.csv
                        (csvfile,
                        row.names=
                        1
                        ))
                    




                        ##	      SampleName    cell   dex albut        Run avgLength Experiment    Sample    BioSample
## SRR1039508 GSM1275862  N61311 untrt untrt SRR1039508       126  SRX384345 SRS508568 SAMN02422669
## SRR1039509 GSM1275863  N61311   trt untrt SRR1039509       126  SRX384346 SRS508567 SAMN02422675
## SRR1039512 GSM1275866 N052611 untrt untrt SRR1039512       126  SRX384349 SRS508571 SAMN02422678
## SRR1039513 GSM1275867 N052611   trt untrt SRR1039513        87  SRX384350 SRS508572 SAMN02422670
## SRR1039516 GSM1275870 N080611 untrt untrt SRR1039516       120  SRX384353 SRS508575 SAMN02422682
## SRR1039517 GSM1275871 N080611   trt untrt SRR1039517       126  SRX384354 SRS508576 SAMN02422673
## SRR1039520 GSM1275874 N061011 untrt untrt SRR1039520       101  SRX384357 SRS508579 SAMN02422683
## SRR1039521 GSM1275875 N061011   trt untrt SRR1039521        98  SRX384358 SRS508580 SAMN02422677
                    


**Note:** here and elsewhere in the workflow, the parentheses () around the entire code of the last line above is an R trick to print the output of the function in addition to saving it to sampleTable. This is equivalent to assigning and then showing the object in two steps:



                        sampleTable <- 
                        read.csv
                        (csvfile,
                        row.names=
                        1
                        )
sampleTable
                    


Once the reads have been aligned, there are a number of tools that can be used to count the number of reads/fragments that can be uniquely assigned to genomic features for each sample. These often take as input SAM/BAM alignment files and a file specifying the genomic features, e.g. a GFF3 or GTF file specifying the gene models.

The following tools can be used generate count matrices: *summarizeOverlaps* ^11^, *featureCounts* ^12^, or *htseq-count* ^13^ ( Table 1).

**Table 1.** Various software which can be used to prepare RNA-seq count matrices.

function	package	framework	output	*DESeq2* input function
*summarizeOverlaps*	*GenomicAlignments*	R/Bioc.	*SummarizedExp.*	*DESeqDataSet*
*featureCounts*	*Rsubread*	R/Bioc.	matrix	*DESeqDataSetFromMatrix*
*htseq-count*	*HTSeq*	Python	files	*DESeqDataSetFromHTSeq*

We now proceed using the *summarizeOverlaps* method of counting. Using the Run column in the sample table, we construct the full paths to the files we want to perform the counting operation on:



                        filenames <- 
                        file.path
                        (dir, 
                        paste0
                        (sampleTable$Run, 
                        "_subset.bam"
                        ))

                        file.exists
                        (filenames)
                    




                        ## [1] TRUE TRUE TRUE TRUE TRUE TRUE TRUE TRUE
                    


We indicate in Bioconductor that these files are BAM files using the *BamFileList* function from the *Rsamtools* package that provides an R interface to BAM files. Here we also specify details about how the BAM files should be treated, e.g., only process 2 million reads at a time. See ?BamFileList for more information.



                        library
                        (
                        "Rsamtools"
                        )
bamfiles <- 
                        BamFileList
                        (filenames, 
                        yieldSize=
                        2000000
                        )
                    


**Note:** make sure that the chromosome names of the genomic features in the annotation you use are consistent with the chromosome names of the reference used for read alignment. Otherwise, the scripts might fail to count any reads to features due to the mismatching names. For example, a common mistake is when the alignment files contain chromosome names in the style of 1 and the gene annotation in the style of chr1, or the other way around. See the *seqlevelsStyle* function in the *GenomeInfoDb* package for solutions. We can check the chromosome names (here called “seqnames”) in the alignment files like so:



                        seqinfo
                        (bamfiles[
                        1
                        ])
                    




                        ## Seqinfo object with 84 sequences from an unspecified genome:
##   seqnames   seqlengths isCircular genome
##   1		 249250621       <NA>   <NA>
##   10          135534747       <NA>   <NA>
##   11          135006516       <NA>   <NA>
##   12          133851895       <NA>   <NA>
##   13          115169878       <NA>   <NA>
##   ...	       ...        ...    ...
##   GL000210.1      27682       <NA>   <NA>
##   GL000231.1      27386       <NA>   <NA>
##   GL000229.1      19913       <NA>   <NA>
##   GL000226.1      15008       <NA>   <NA>
##   GL000207.1       4262       <NA>   <NA>
                    


### Defining gene models

Next, we need to read in the gene model that will be used for counting reads/fragments. We will read the gene model from an Ensembl GTF file ^8^, using *makeTxDbFromGFF* from the *GenomicFeatures* package. GTF files can be downloaded from Ensembl’s FTP site or other gene model repositories. A *TxDb* object is a database that can be used to generate a variety of range-based objects, such as exons, transcripts, and genes. We want to make a list of exons grouped by gene for counting read/fragments.

There are other options for constructing a *TxDb*. For the *known genes* track from the UCSC Genome Browser ^14^, one can use the pre-built Transcript DataBase: *TxDb.Hsapiens.UCSC.hg19.knownGene*. If the annotation file is accessible from *AnnotationHub* (as is the case for the Ensembl genes), a pre-scanned GTF file can be imported using *makeTxDbFromGRanges*. Finally, the *makeTxDbFromBiomart* function can be used to automatically pull a gene model from Biomart using *biomaRt* ^15^.

Here we will demonstrate loading from a GTF file:



                        library
                        (
                        "GenomicFeatures"
                        )
                    


We indicate that none of our sequences (chromosomes) are circular using a 0-length character vector.



                        gtffile <- 
                        file.path
                        (dir,
                        “Homo_sapiens.GRCh37.75_subset.gtf"
                        )
(txdb <- 
                        makeTxDbFromGFF
                        (gtffile, 
                        format=
                        “gtf"
                        , 
                        circ_seqs=character
                        ()))
                    




                        ## TxDb object:
## # Db type: TxDb
## # Supporting package: GenomicFeatures
## # Data source: /Users/michael/Library/R/3.2/library/airway/extdata/Homo_sapiens.GRCh37.75_subset.gtf
## # Organism: NA
## # miRBase build ID: NA
## # Genome: NA
## # transcript_nrow: 65
## # exon_nrow: 279
## # cds_nrow: 158
## # Db created by: GenomicFeatures package from Bioconductor
## # Creation time: 2015-09-09 14:48:56 -0400 (Wed, 09 Sep 2015)
## # GenomicFeatures version at creation time: 1.20.3
## # RSQLite version at creation time: 1.0.0
## # DBSCHEMAVERSION: 1.1
                    


The following line produces a *GRangesList* of all the exons grouped by gene ^11^. Each element of the list is a *GRanges* object of the exons for a gene.



                        (ebg <- 
                        exonsBy
                        (txdb, 
                        by=
                        “gene"
                        ))
                    




                        ## GRangesList object of length 20:
## $ENSG00000009724
## GRanges object with 18 ranges and 2 metadata columns:
##	  seqnames		 ranges strand	 |   exon_id	   exon_name
##	     <Rle>	      <IRanges>	 <Rle>	 | <integer>	 <character>
##    [1]	 1 [11086580, 11087705]	     -	 |	  98 ENSE00000818830
##    [2]	 1 [11090233, 11090307]	     -	 |	  99 ENSE00000472123
##    [3]	 1 [11090805, 11090939]	     -	 |	 100 ENSE00000743084
##    [4]	 1 [11094885, 11094963]	     -	 |	 101 ENSE00000743085
##    [5]	 1 [11097750, 11097868]	     -	 |	 103 ENSE00003520086
##    ...      ...		    ...	   ... ...	 ...		 ...
##   [14]	 1 [11106948, 11107176]	     -	 |	 111 ENSE00003467404
##   [15]	 1 [11106948, 11107176]	     -	 |	 112 ENSE00003489217
##   [16]	 1 [11107260, 11107280]	     -	 |	 113 ENSE00001833377
##   [17]	 1 [11107260, 11107284]	     -	 |	 114 ENSE00001472289
##   [18]	 1 [11107260, 11107290]	     -	 |	 115 ENSE00001881401
##								
## ...
## <19 more elements>
## -------
## seqinfo: 1 sequence from an unspecified genome; no seqlengths
                    


### Read counting step

After these preparations, the actual counting is easy. The function *summarizeOverlaps* from the *GenomicAlignments* package will do this. This produces a *SummarizedExperiment* object that contains a variety of information about the experiment, and will be described in more detail below.

**Note:** If it is desired to perform counting using multiple cores, one can use the *register* and *MulticoreParam* or *SnowParam* functions from the *BiocParallel* package before the counting call below. Expect that the summarizeOverlaps call will take at least 30 minutes per file for a human RNA-seq file with 30 million aligned reads. By sending the files to separate cores, one can speed up the entire counting process.



                        library
                        (
                        "GenomicAlignments"
                        )

                        library
                        (
                        "BiocParallel"
                        )
                    


Here we specify to use one core, not multiple cores. We could have also skipped this line and the counting step would run in serial.



                        register
                        (
                        SerialParam
                        ())
                    


The following call creates the *SummarizedExperiment* object with counts:



                        se <- 
                        summarizeOverlaps
                        (
                        features=
                        ebg, 
                        reads=
                        bamfiles,
			  
                        mode=
                        “Union"
                        ,
			  
                        singleEnd=
                        FALSE
                        ,
			  
                        ignore.strand=
                        TRUE
                        ,
			  
                        fragments=
                        TRUE 
                        )
                    


**Figure 1.**
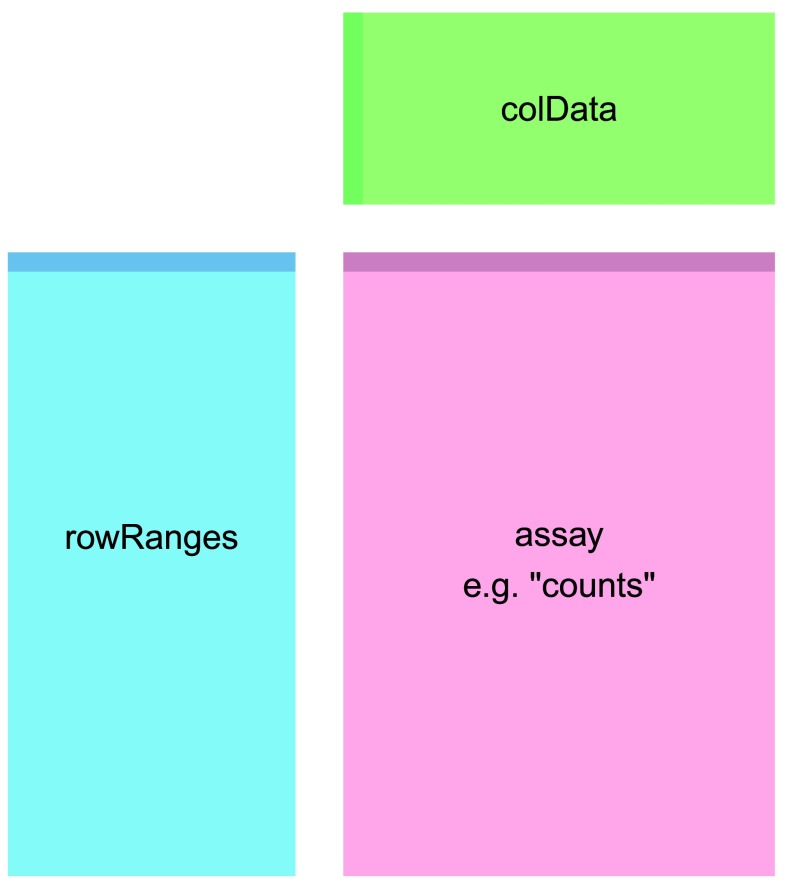
The component parts of a *SummarizedExperiment* object. The assay (pink block) contains the matrix of counts, the rowRanges (blue block) contains information about the genomic ranges and the colData (green block) contains information about the samples. The highlighted line in each block represents the first row (note that the first row of colData lines up with the first column of the assay).

We specify a number of arguments besides the features and the reads. The mode argument describes what kind of read overlaps will be counted. These modes are shown in Figure 1 of the *Counting reads with summarizeOverlaps* vignette for the *GenomicAlignments* package. Note that fragments will be counted only once to each gene, even if they overlap multiple exons of a gene which may themselves be overlapping. Setting singleEnd to FALSE indicates that the experiment produced paired-end reads, and we want to count a pair of reads (a fragment) only once toward the count for a gene.

In order to produce correct counts, it is important to know if the RNA-seq experiment was strand-specific or not. This experiment was not strand-specific so we set ignore.strand to TRUE. The fragments argument can be used when singleEnd=FALSE to specify if unpaired reads should be counted (yes if fragments=TRUE).

### SummarizedExperiment

The *SummarizedExperiment* container is diagrammed in Figure 1 and discussed in the latest Bioconductor paper ^16^. In our case we have created a single matrix named “counts” that contains the fragment counts for each gene and sample, which is stored in assay. It is also possible to store multiple matrices, accessed with assays. The rowRanges for our object is the *GRangesList* we used for counting (one *GRanges* of exons for each row of the count matrix). The component parts of the *SummarizedExperiment* are accessed with an R function of the same name: assay (or assays), rowRanges and colData.

This example code above actually only counted a small subset of fragments from the original experiment. Nevertheless, we can still investigate the resulting *SummarizedExperiment* by looking at the counts in the assay slot, the phenotypic data about the samples in colData slot (in this case an empty *DataFrame*), and the data about the genes in the rowRanges slot.



                        se
                    




                        ## class: SummarizedExperiment
## dim: 20 8
## exptData(0):
## assays(1): counts
## rownames(20): ENSG00000009724 ENSG00000116649 ... ENSG00000271794 ENSG00000271895
## rowRanges metadata column names(0):
## colnames(8): SRR1039508_subset.bam SRR1039509_subset.bam ... SRR1039520_subset.bam SRR1039521_subset.bam
## colData names(0):
                    




                        dim
                        (se)
                    




                        ## [1] 20 8
                    




                        assayNames
                        (se)
                    




                        ## [1] "counts"
                    




                        head
                        (
                        assay
                        (se), 
                        3
                        )
                    




                        ##		   SRR1039508_subset.bam	SRR1039509_subset.bam	SRR1039512_subset.bam
## ENSG00000009724		      38		           28		           66
## ENSG00000116649		    1004		         1255		         1122
## ENSG00000120942		     218		          256		          233
##		   SRR1039513_subset.bam	SRR1039516_subset.bam	SRR1039517_subset.bam
## ENSG00000009724		      24		           42		           41
## ENSG00000116649		    1313		         1100		         1879
## ENSG00000120942		     252		          269		          465
##		   SRR1039520_subset.bam	SRR1039521_subset.bam	
## ENSG00000009724		      47		           36	
## ENSG00000116649		     745		         1536	
## ENSG00000120942		     207		          400
                    




                        colSums
                        (
                        assay
                        (se))
                    




                        ## SRR1039508_subset.bam SRR1039509_subset.bam SRR1039512_subset.bam SRR1039513_subset.bam
##		    6478		  6501		        7699		      6801
## SRR1039516_subset.bam SRR1039517_subset.bam SRR1039520_subset.bam SRR1039521_subset.bam
##		    8009		 10849		        5254		      9168
                    


The rowRanges, when printed, only shows the first *GRanges*, and tells us there are 19 more elements:



                        rowRanges
                        (se)
                    




                        ## GRangesList object of length 20:
## $ENSG00000009724
## GRanges object with 18 ranges and 2 metadata columns:
##	  seqnames		 ranges strand	 |   exon_id	   exon_name
##	     <Rle>	      <IRanges>	 <Rle>	 | <integer>	 <character>
##    [1]	 1 [11086580, 11087705]	     -	 |	  98 ENSE00000818830
##    [2]	 1 [11090233, 11090307]	     -	 |	  99 ENSE00000472123
##    [3]	 1 [11090805, 11090939]	     -	 |	 100 ENSE00000743084
##    [4]	 1 [11094885, 11094963]	     -	 |	 101 ENSE00000743085
##    [5]	 1 [11097750, 11097868]	     -	 |	 103 ENSE00003520086
##    ...      ...		    ...	   ... ...	 ...		 ...
##   [14]	 1 [11106948, 11107176]	     -	 |	 111 ENSE00003467404
##   [15]	 1 [11106948, 11107176]	     -	 |	 112 ENSE00003489217
##   [16]	 1 [11107260, 11107280]	     -	 |	 113 ENSE00001833377
##   [17]	 1 [11107260, 11107284]	     -	 |	 114 ENSE00001472289
##   [18]	 1 [11107260, 11107290]	     -	 |	 115 ENSE00001881401
##								
## ...
## <19 more elements>
## -------
## seqinfo: 1 sequence from an unspecified genome; no seqlengths
                    


The rowRanges also contains metadata about the construction of the gene model in the metadata slot. Here we use a helpful R function, str, to display the metadata compactly:



                        str
                        (
                        metadata
                        (
                        rowRanges
                        (se)))
                    




                        ## List of 1
##  $ genomeInfo:List of 14
##   ..$ Db type				 : chr "TxDb"
##   ..$ Supporting package		  	 : chr "GenomicFeatures"
##   ..$ Data source				 : chr "/Users/michael/Library/R/3.2/library/airway/extd
##   ..$ Organism				 : chr NA
##   ..$ miRBase build ID		 	 : chr NA
##   ..$ Genome					 : chr NA
##   ..$ transcript_nrow		 	 : chr "65"
##   ..$ exon_nrow				 : chr "279"
##   ..$ cds_nrow				 : chr "158"
##   ..$ Db created by			 	 : chr "GenomicFeatures package from Bioconductor"
##   ..$ Creation time				 : chr "2015-09-09 14:48:56 -0400 (Wed, 09 Sep 2015)"
##   ..$ GenomicFeatures version at creation time: chr "1.20.3"
##   ..$ RSQLite version at creation time	 : chr "1.0.0"
##   ..$ DBSCHEMAVERSION	    		 : chr "1.1"
                    


The colData:



                        colData
                        (se)
                    




                        ## DataFrame with 8 rows and 0 columns
                    


The colData slot, so far empty, should contain all the metadata. Because we used a column of sampleTable to produce the bamfiles vector, we know the columns of se are in the same order as the rows of sampleTable. We can assign the sampleTable as the colData of the summarized experiment, by converting it into a *DataFrame* and using the assignment function:



                        (
                        colData
                        (se) <- 
                        DataFrame
                        (sampleTable))
                    




                        ## DataFrame with 8 rows and 9 columns
##	      SampleName     cell      dex    albut	   Run avgLength  Experiment	Sample
##	        <factor> <factor> <factor> <factor>   <factor> <integer>    <factor>  <factor>
## SRR1039508 GSM1275862   N61311    untrt    untrt SRR1039508	     126   SRX384345 SRS508568
## SRR1039509 GSM1275863   N61311      trt    untrt SRR1039509	     126   SRX384346 SRS508567
## SRR1039512 GSM1275866  N052611    untrt    untrt SRR1039512	     126   SRX384349 SRS508571
## SRR1039513 GSM1275867  N052611      trt    untrt SRR1039513	      87   SRX384350 SRS508572
## SRR1039516 GSM1275870  N080611    untrt    untrt SRR1039516	     120   SRX384353 SRS508575
## SRR1039517 GSM1275871  N080611      trt    untrt SRR1039517	     126   SRX384354 SRS508576
## SRR1039520 GSM1275874  N061011    untrt    untrt SRR1039520	     101   SRX384357 SRS508579
## SRR1039521 GSM1275875  N061011      trt    untrt SRR1039521	      98   SRX384358 SRS508580
##		 BioSample	
##		  <factor>
## SRR1039508 SAMN02422669	
## SRR1039509 SAMN02422675	
## SRR1039512 SAMN02422678	
## SRR1039513 SAMN02422670	
## SRR1039516 SAMN02422682	
## SRR1039517 SAMN02422673	
## SRR1039520 SAMN02422683	
## SRR1039521 SAMN02422677
                    


### Branching point

At this point, we have counted the fragments which overlap the genes in the gene model we specified. This is a branching point where we could use a variety of Bioconductor packages for exploration and differential expression of the count data, including *edgeR* ^3^, *limma* with the voom method ^4^, *DSS* ^5^, *EBSeq* ^6^ and *BaySeq* ^7^. We will continue, using *DESeq2* ^2^. The *SummarizedExperiment* object is all we need to start our analysis. In the following section we will show how to use it to create the data object used by *DESeq2*.

## The *DESeqDataSet*, sample information, and the design formula

Bioconductor software packages often define and use a custom class for storing data that makes sure that all the needed data slots are consistently provided and fulfill the requirements. In addition, Bioconductor has general data classes (such as the *SummarizedExperiment*) that can be used to move data between packages. Additionally, the core Bioconductor classes provide useful functionality: for example, subsetting or reordering the rows or columns of a *SummarizedExperiment* automatically subsets or reorders the associated *rowRanges* and *colData*, which can help to prevent accidental sample swaps that would otherwise lead to spurious results. With *SummarizedExperiment* this is all taken care of behind the scenes.

In *DESeq2*, the custom class is called *DESeqDataSet*. It is built on top of the *SummarizedExperiment* class, and it is easy to convert *SummarizedExperiment* objects into *DESeqDataSet* objects, which we show below. One of the two main differences is that the assay slot is instead accessed using the *counts* accessor function, and the *DESeqDataSet* class enforces that the values in this matrix are non-negative integers.

A second difference is that the *DESeqDataSet* has an associated *design formula*. The experimental design is specified at the beginning of the analysis, as it will inform many of the *DESeq2* functions how to treat the samples in the analysis (one exception is the size factor estimation, i.e., the adjustment for differing library sizes, which does not depend on the design formula). The design formula tells which columns in the sample information table ( colData) specify the experimental design and how these factors should be used in the analysis.

The simplest design formula for differential expression would be ~ condition, where condition is a column in colData(dds) that specifies which of two (or more groups) the samples belong to. For the airway experiment, we will specify ~ cell + dex meaning that we want to test for the effect of dexamethasone ( dex) controlling for the effect of different cell line ( cell). We can see each of the columns just using the $ directly on the *SummarizedExperiment* or *DESeqDataSet*:



                    se$cell
                




                    ## [1] N61311  N61311  N052611 N052611 N080611 N080611 N061011 N061011
## Levels: N052611 N061011 N080611 N61311
                




                    se$dex
                




                    ## [1] untrt trt   untrt trt   untrt trt   untrt trt
## Levels: trt untrt
                


**Note:** it is prefered in R that the first level of a factor be the reference level (e.g. control, or untreated samples), so we can *relevel* the dex factor like so:



                    se$dex <- 
                    relevel
                    (se$dex, 
                    "untrt"
                    )
se$dex
                




                    ## [1] untrt trt   untrt trt   untrt trt   untrt trt
## Levels: untrt trt
                


For running *DESeq2* models, you can use R’s formula notation to express any fixed-effects experimental design. Note that *DESeq2* uses the same formula notation as, for instance, the *lm* function of base R. If the research aim is to determine for which genes the effect of treatment is different across groups, then interaction terms can be included and tested using a design such as ~ group + treatment + group:treatment. See the manual page for ?results for more examples. We will show how to use an interaction term to test for condition-specific changes over time in a time course example below.

In the following sections, we will demonstrate the construction of the *DESeqDataSet* from two starting points:

- from a *SummarizedExperiment* object
- from a count matrix and a sample information table

For a full example of using the *HTSeq* Python package for read counting, please see the *pasilla* vignette. For an example of generating the *DESeqDataSet* from files produced by *htseq-count*, please see the *DESeq2* vignette.

## Starting from *SummarizedExperiment*

We now use R’s *data* command to load a prepared *SummarizedExperiment* that was generated from the publicly available sequencing data files associated with the Himes *et al.* ^1^ paper, described above. The steps we used to produce this object were equivalent to those you worked through in the previous sections, except that we used all the reads and all the genes. For more details on the exact steps used to create this object, type vignette( "airway") into your R session.



                    data
                    (
                    "airway"
                    )
se <- airway
                


Again, we want to specify that untrt is the reference level for the dex variable:



                    se$dex <- 
                    relevel
                    (se$dex, 
                    "untrt"
                    )
se$dex
                




                    ## [1] untrt trt   untrt trt   untrt trt   untrt trt
## Levels: untrt trt
                


We can quickly check the millions of fragments that uniquely aligned to the genes (the second argument of *round* tells how many decimal points to keep).



                    round
                    ( 
                    colSums
                    (
                    assay
                    (se)) / 
                    1e6
                    , 
                    1 
                    )
                




                    ## SRR1039508 SRR1039509 SRR1039512 SRR1039513 SRR1039516 SRR1039517 SRR1039520 SRR1039521
##       20.6       18.8       25.3       15.2       24.4       30.8       19.1       21.2
                


Supposing we have constructed a *SummarizedExperiment* using one of the methods described in the previous section, we now need to make sure that the object contains all the necessary information about the samples, i.e., a table with metadata on the count matrix’s columns stored in the colData slot:



                    colData
                    (se)
                




                    ## DataFrame with 8 rows and 9 columns
##	      SampleName     cell      dex    albut	   Run avgLength  Experiment	Sample
##	        <factor> <factor> <factor> <factor>   <factor> <integer>    <factor>  <factor>
## SRR1039508 GSM1275862   N61311    untrt    untrt SRR1039508	     126   SRX384345 SRS508568
## SRR1039509 GSM1275863   N61311      trt    untrt SRR1039509	     126   SRX384346 SRS508567
## SRR1039512 GSM1275866  N052611    untrt    untrt SRR1039512	     126   SRX384349 SRS508571
## SRR1039513 GSM1275867  N052611      trt    untrt SRR1039513	      87   SRX384350 SRS508572
## SRR1039516 GSM1275870  N080611    untrt    untrt SRR1039516	     120   SRX384353 SRS508575
## SRR1039517 GSM1275871  N080611      trt    untrt SRR1039517	     126   SRX384354 SRS508576
## SRR1039520 GSM1275874  N061011    untrt    untrt SRR1039520	     101   SRX384357 SRS508579
## SRR1039521 GSM1275875  N061011      trt    untrt SRR1039521	      98   SRX384358 SRS508580
##		 BioSample	
##		  <factor>
## SRR1039508 SAMN02422669	
## SRR1039509 SAMN02422675	
## SRR1039512 SAMN02422678	
## SRR1039513 SAMN02422670	
## SRR1039516 SAMN02422682	
## SRR1039517 SAMN02422673	
## SRR1039520 SAMN02422683	
## SRR1039521 SAMN02422677
                


Here we see that this object already contains an informative colData slot – because we have already prepared it for you, as described in the *airway* vignette. However, when you work with your own data, you will have to add the pertinent sample/phenotypic information for the experiment at this stage. We highly recommend keeping this information in a comma-separated value (CSV) or tab-separated value (TSV) file, which can be exported from an Excel spreadsheet, and the assign this to the colData slot, making sure that the rows correspond to the columns of the *SummarizedExperiment*. We made sure of this correspondence earlier by specifying the BAM files using a column of the sample table.

Once we have our fully annotated *SummarizedExperiment* object, we can construct a *DESeqDataSet* object from it that will then form the starting point of the analysis. We add an appropriate design for the analysis:



                    library
                    (
                    "DESeq2"
                    )
                




                    dds <- 
                    DESeqDataSet
                    (se, 
                    design = 
                    ~ cell + dex)
                


If we only wanted to perform transformations and exploratory data analysis (as explained later in this workflow) we could use a ~ 1 for the design, but we would need to remember to substitute a real design, e.g. ~ condition, before we run *DESeq* for differential testing or else we would only be testing the intercept.

### Starting from count matrices

In this section, we will show how to build an *DESeqDataSet* supposing we only have a count matrix and a table of sample information.

**Note:** if you have prepared a *SummarizedExperiment* you should skip this section. While the previous section would be used to construct a *DESeqDataSet* from a *SummarizedExperiment*, here we first extract the individual object (count matrix and sample info) from the *SummarizedExperiment* in order to build it back up into a new object – only for demonstration purposes. In practice, the count matrix would either be read in from a file or perhaps generated by an R function like *featureCounts* from the *Rsubread* package ^12^.

The information in a *SummarizedExperiment* object can be accessed with accessor functions. For example, to see the actual data, i.e., here, the fragment counts, we use the *assay* function. (The *head* function restricts the output to the first few lines.)



                        countdata <- 
                        assay
                        (se)

                        head
                        (countdata, 
                        3
                        )
                    




                        ##		  SRR1039508 SRR1039509 SRR1039512 SRR1039513 SRR1039516 SRR1039517 SRR1039520
## ENSG00000000003	 679	    448	       873        408       1138       1047	   770
## ENSG00000000005	   0	      0	   	 0	    0	       0	  0	     0
## ENSG00000000419	 467	    515	       621        365        587        799	   417
##		  SRR1039521	
## ENSG00000000003	 572	
## ENSG00000000005	   0	
## ENSG00000000419	 508
                    


In this count matrix, each row represents an Ensembl gene, each column a sequenced RNA library, and the values give the raw numbers of fragments that were uniquely assigned to the respective gene in each library. We also have information on each of the samples (the columns of the count matrix). If you’ve counted reads with some other software, it is very important to check that the columns of the count matrix correspond to the rows of the sample information table.



                        coldata <- 
                        colData
                        (se)
                    


We now have all the ingredients to prepare our data object in a form that is suitable for analysis, namely:

- countdata: a table with the fragment counts
- coldata: a table with information about the samples

To now construct the *DESeqDataSet* object from the matrix of counts and the sample information table, we use:



                        (ddsMat <- 
                        DESeqDataSetFromMatrix
                        (
                        countData = 
                        countdata,
                                      
                        colData = 
                        coldata,
                                      
                        design = 
                        ~ cell + dex))
                    




                        ## class: DESeqDataSet
## dim: 64102 8
## exptData(0):
## assays(1): counts
## rownames(64102): ENSG00000000003 ENSG00000000005 ... LRG_98 LRG_99
## rowRanges metadata column names(0):
## colnames(8): SRR1039508 SRR1039509 ... SRR1039520 SRR1039521
## colData names(9): SampleName cell ... Sample BioSample
                    


We will continue with the object generated from the *SummarizedExperiment* section.

## Exploratory analysis and visualization

There are two separate paths in this workflow; the one we will see first involves *transformations of the counts* in order to visually explore sample relationships. In the second part, we will go back to the original raw counts for *statistical testing*. This is critical because the statistical testing methods rely on original count data (not scaled or transformed) for calculating the precision of measurements.

### Pre-filtering the dataset

Our count matrix with our *DESeqDataSet* contains many rows with only zeros, and additionally many rows with only a few fragments total. In order to reduce the size of the object, and to increase the speed of our functions, we can remove the rows that have no or nearly no information about the amount of gene expression. Here we remove rows of the *DESeqDataSet* that have no counts, or only a single count across all samples:



                        nrow
                        (dds)
                    




                        ## [1] 64102
                    




                        dds <- dds[ 
                        rowSums
                        (
                        counts
                        (dds)) > 
                        1
                        , ]

                        nrow
                        (dds)
                    




                        ## [1] 29391
                    


### The rlog transformation

Many common statistical methods for exploratory analysis of multidimensional data, for example clustering and *principal components analysis* (PCA), work best for data that generally has the same range of variance at different ranges of the mean values. When the expected amount of variance is approximately the same across different mean values, the data is said to be *homoskedastic*. For RNA-seq raw counts, however, the variance grows with the mean. For example, if one performs PCA directly on a matrix of size-factor-normalized read counts, the result typically depends only on the few most strongly expressed genes because they show the largest absolute differences between samples. A simple and often used strategy to avoid this is to take the logarithm of the normalized count values plus a small pseudocount; however, now the genes with the very lowest counts will tend to dominate the results because, due to the strong Poisson noise inherent to small count values, and the fact that the logarithm amplifies differences for the smallest values, these low count genes will show the strongest relative differences between samples.

As a solution, *DESeq2* offers transformations for count data that stabilize the variance across the mean. One such transformation is the *regularized-logarithm transformation* or *rlog* ^2^. For genes with high counts, the rlog transformation will give similar result to the ordinary log2 transformation of normalized counts. For genes with lower counts, however, the values are shrunken towards the genes’ averages across all samples. Using an empirical Bayesian prior on inter-sample differences in the form of a *ridge penalty*, the rlog-transformed data then becomes approximately *homoskedastic*, and can be used directly for computing distances between samples and making PCA plots. Another transformation, the *variance stabilizing transformation* ^17^, is discussed alongside the *rlog* in the *DESeq2* vignette.

**Note:** the rlog transformation is provided for applications *other* than differential testing. For differential testing we recommend the *DESeq* function applied to raw counts, as described later in this workflow, which also takes into account the dependence of the variance of counts on the mean value during the dispersion estimation step.

The function *rlog* returns a *SummarizedExperiment* object that contains the rlog-transformed values in its *assay* slot.



                        rld <- 
                        rlog
                        (dds, 
                        blind=
                        FALSE
                        )

                        head
                        (
                        assay
                        (rld), 
                        3
                        )
                    




                        ##		   SRR1039508 SRR1039509 SRR1039512 SRR1039513 SRR1039516 SRR1039517 SRR1039520
## ENSG00000000003   9.385536   9.051592   9.517044   9.284930   9.839980   9.530510   9.663767
## ENSG00000000419   8.868967   9.138776   9.036191   9.075538   8.971927   9.132297   8.860846
## ENSG00000000457   7.962223   7.881317   7.823335   7.921887   7.750083   7.886432   7.957928
##		   SRR1039521	
## ENSG00000000003   9.277281	
## ENSG00000000419   9.061085	
## ENSG00000000457   7.912412
                    


We specify blind=FALSE, which means that differences between cell lines and treatment should not add to the variance-mean profile of the experiment. However, the experimental design is not used directly in the transformation, only in estimating the global amount of variability in the counts. For a fully *unsupervised* transformation, one can set blind=TRUE (which is the default).

**Note:** for large datasets (hundreds of samples), the variance stabilizing transformation will be faster to compute.

**Figure 2.**
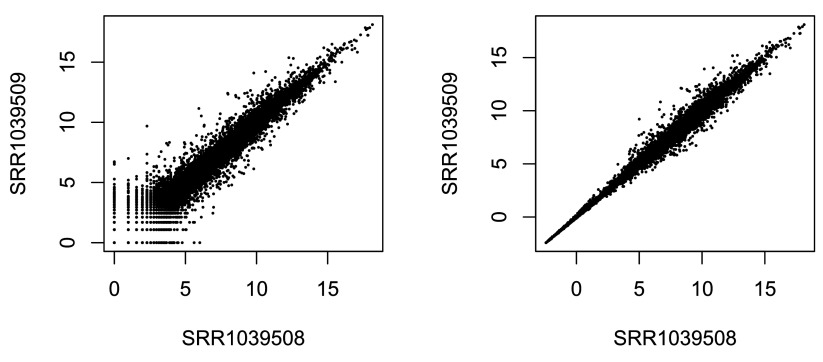
Scatterplot of transformed counts from two samples. Shown are scatterplots using the log2 transform of normalized counts (left side) and using the rlog (right side).



To show the effect of the transformation, in Figure 2 we plot the first sample against the second, first simply using the *log2* function (after adding 1, to avoid taking the log of zero), and then using the rlog-transformed values. For the *log2* approach, we need to first estimate *size factors* to account for sequencing depth, and then specify normalized=TRUE. Sequencing depth correction is done automatically for the *rlog* method (and for *varianceStabilizingTransformation*).

                        par
                        ( 
                        mfrow = c
                        ( 
                        1
                        , 
                        2 
                        ) )
dds <- 
                        estimateSizeFactors
                        (dds)

                        plot
                        (
                        log2
                        (
                        counts
                        (dds, 
                        normalized=
                        TRUE
                        )[,
                        1
                        :
                        2
                        ] + 
                        1
                        ),
     
                        pch=
                        16
                        , 
                        cex=
                        0.3
                        )

                        plot
                        (
                        assay
                        (rld)[,
                        1
                        :
                        2
                        ],
     
                        pch=
                        16
                        , 
                        cex=
                        0.3
                        )
                    


We can see how genes with low counts (bottom left-hand corner) seem to be excessively variable on the ordinary logarithmic scale, while the rlog transform compresses differences for the low count genes for which the data provide little information about differential expression.

### Sample distances

A useful first step in an RNA-seq analysis is often to assess overall similarity between samples: Which samples are similar to each other, which are different? Does this fit to the expectation from the experiment’s design?

We use the R function *dist* to calculate the Euclidean distance between samples. To ensure we have a roughly equal contribution from all genes, we use it on the rlog-transformed data. We need to transpose the matrix of values using *t*, because the *dist* function expects the different samples to be rows of its argument, and different dimensions (here, genes) to be columns.



                        sampleDists <- 
                        dist
                        ( 
                        t
                        ( 
                        assay
                        (rld) ) )
sampleDists
                    




                        ##	      SRR1039508 SRR1039509 SRR1039512 SRR1039513 SRR1039516 SRR1039517 SRR1039520
## SRR1039509   46.25524						
## SRR1039512   39.94490   55.67572					
## SRR1039513   63.36642   45.19462   49.30007				
## SRR1039516   45.28129   59.89304   44.32383   64.54450			
## SRR1039517   65.34730   52.25475   60.05523   50.64861   48.05714		
## SRR1039520   40.20215   58.19904   37.35413   59.19401   47.15396   64.44641	
## SRR1039521   64.09339   45.70177   58.59277   37.10803   66.36711   53.09669   50.72310
                    


**Figure 3.**
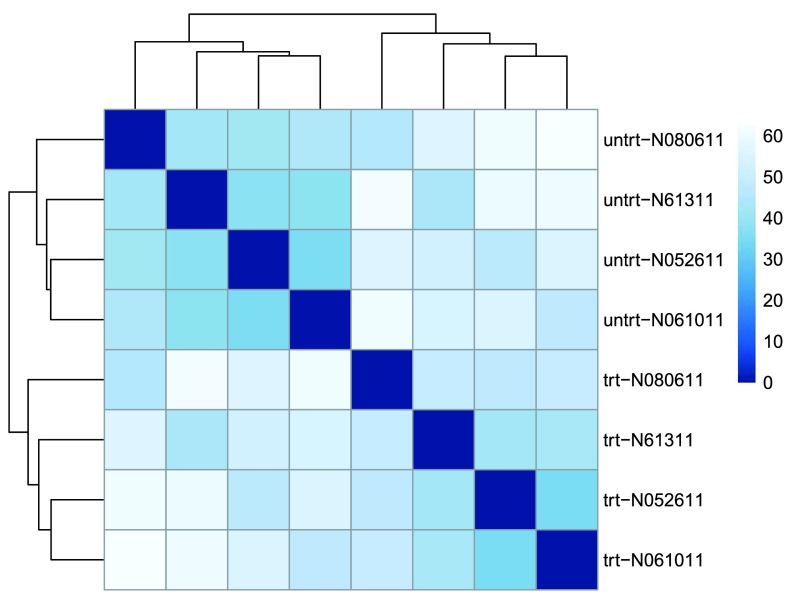
Heatmap of sample-to-sample distances using the rlog-transformed values.



We visualize the distances in a heatmap in Figure 3, using the function *pheatmap* from the *pheatmap* package.

                        library
                        (
                        "pheatmap"
                        )

                        library
                        (
                        "RColorBrewer"
                        )
                    


In order to plot the sample distance matrix with the rows/columns arranged by the distances in our distance matrix, we manually provide sampleDists to the clustering_distance argument of the *pheatmap* function. Otherwise the *pheatmap* function would assume that the matrix contains the data values themselves, and would calculate distances between the rows/columns of the distance matrix, which is not desired. We also manually specify a blue color palette using the *colorRampPalette* function from the *RColorBrewer* package.



                        sampleDistMatrix <- 
                        as.matrix
                        ( sampleDists )

                        rownames
                        (sampleDistMatrix) <- 
                        paste
                        ( rld$dex, rld$cell, 
                        sep=
                        "-" 
                        )

                        colnames
                        (sampleDistMatrix) <- 
                        NULL

                        colors <- 
                        colorRampPalette
                        ( 
                        rev
                        (
                        brewer.pal
                        (
                        9
                        , 
                        "Blues"
                        )) )(
                        255
                        )

                        pheatmap
                        (sampleDistMatrix,
	  
                        clustering_distance_rows=
                        sampleDists,
	  
                        clustering_distance_cols=
                        sampleDists,
	  
                        col=
                        colors)
                    


Note that we have changed the row names of the distance matrix to contain treatment type and patient number instead of sample ID, so that we have all this information in view when looking at the heatmap.

Another option for calculating sample distances is to use the *Poisson Distance* ^18^, implemented in the *PoiClaClu* package. This measure of dissimilarity between counts also takes the inherent variance structure of counts into consideration when calculating the distances between samples. The *PoissonDistance* function takes the original count matrix (not normalized) with samples as rows instead of columns, so we need to transpose the counts in dds.



                        library
                        (
                        "PoiClaClu"
                        )

                        poisd <- 
                        PoissonDistance
                        (
                        t
                        (
                        counts
                        (dds)))
                    


**Figure 4.**
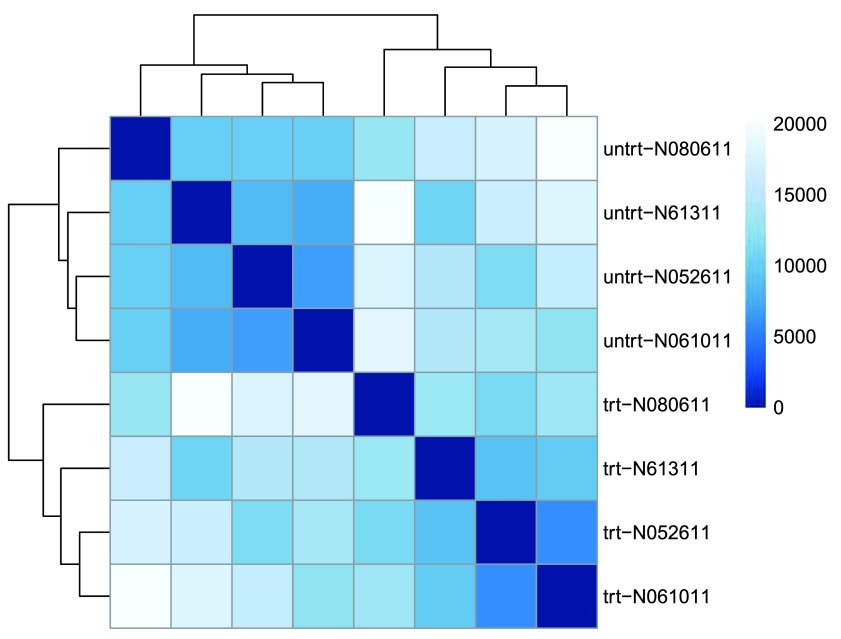
Heatmap of sample-to-sample distances using the *Poisson Distance*.



We plot the *Poisson Distance* heatmap in Figure 4.

                        samplePoisDistMatrix <- 
                        as.matrix
                        ( poisd$dd )

                        rownames
                        (samplePoisDistMatrix) <- 
                        paste
                        ( rld$dex, rld$cell, 
                        sep=
                        "-" 
                        )

                        colnames
                        (samplePoisDistMatrix) <- 
                        NULL

                        pheatmap
                        (samplePoisDistMatrix,
	  
                        clustering_distance_rows=
                        poisd$dd,
	  
                        clustering_distance_cols=
                        poisd$dd,
	  
                        col=
                        colors)
                    


### PCA plot

**Figure 5.**
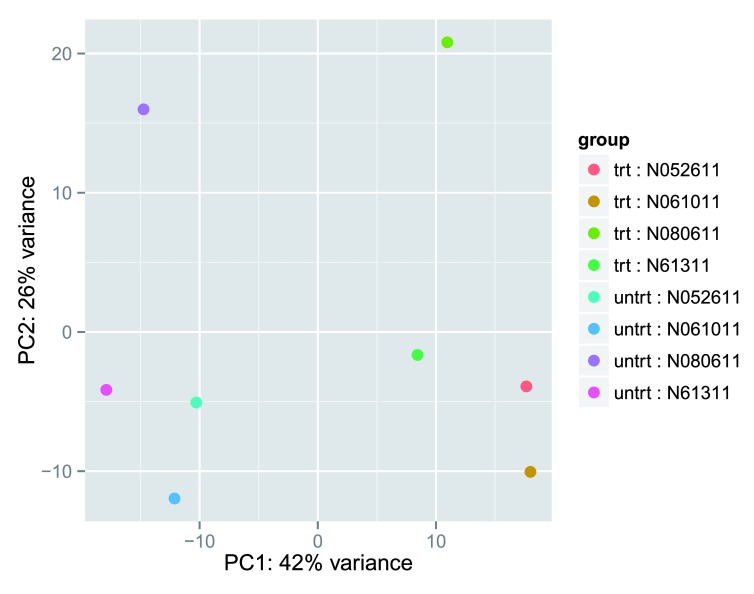
PCA plot using the rlog-transformed values. Each unique combination of treatment and cell line is given its own color.




Another way to visualize sample-to-sample distances is a principal components analysis (PCA). In this ordination method, the data points (here, the samples) are projected onto the 2D plane such that they spread out in the two directions that explain most of the differences ( Figure 5). The x-axis is the direction that separates the data points the most. The values of the samples in this direction are written *PC1*. The y-axis is a direction (it must be *orthogonal* to the first direction) that separates the data the second most. The values of the samples in this direction are written *PC2*. The percent of the total variance that is contained in the direction is printed in the axis label. Note that these percentages do not add to 100%, because there are more dimensions that contain the remaining variance (although each of these remaining dimensions will explain less than the two that we see).

                        plotPCA
                        (rld,  
                        intgroup = c
                        (
                        "dex"
                        , 
                        "cell"
                        ))
                    


Here, we have used the function *plotPCA* that comes with *DESeq2*. The two terms specified by intgroup are the interesting groups for labeling the samples; they tell the function to use them to choose colors. We can also build the PCA plot from scratch using the *ggplot2* package ^19^. This is done by asking the *plotPCA* function to return the data used for plotting rather than building the plot. See the *ggplot2* documentation for more details on using *ggplot*.



                        (data <- 
                        plotPCA
                        (rld, 
                        intgroup = c
                        ( 
                        "dex"
                        , 
                        "cell"
                        ), 
                        returnData=
                        TRUE
                        ))
                    




                        ##		    PC1	       PC2	     group   dex    cell       name
## SRR1039508 -17.88882	 -4.157888  untrt : N61311 untrt  N61311 SRR1039508
## SRR1039509	8.43675  -1.650879    trt : N61311   trt  N61311 SRR1039509
## SRR1039512 -10.27798	 -5.066577 untrt : N052611 untrt N052611 SRR1039512
## SRR1039513  17.64271	 -3.910902   trt : N052611   trt N052611 SRR1039513
## SRR1039516 -14.74069	 15.990031 untrt : N080611 untrt N080611 SRR1039516
## SRR1039517  10.95638	 20.806181   trt : N080611   trt N080611 SRR1039517
## SRR1039520 -12.12010 -11.962545 untrt : N061011 untrt N061011 SRR1039520
## SRR1039521  17.99175 -10.047421   trt : N061011   trt N061011 SRR1039521
                    




                        percentVar <- 
                        round
                        (
                        100 
                        * 
                        attr
                        (data, 
                        "percentVar"
                        ))
                    


**Figure 6.**
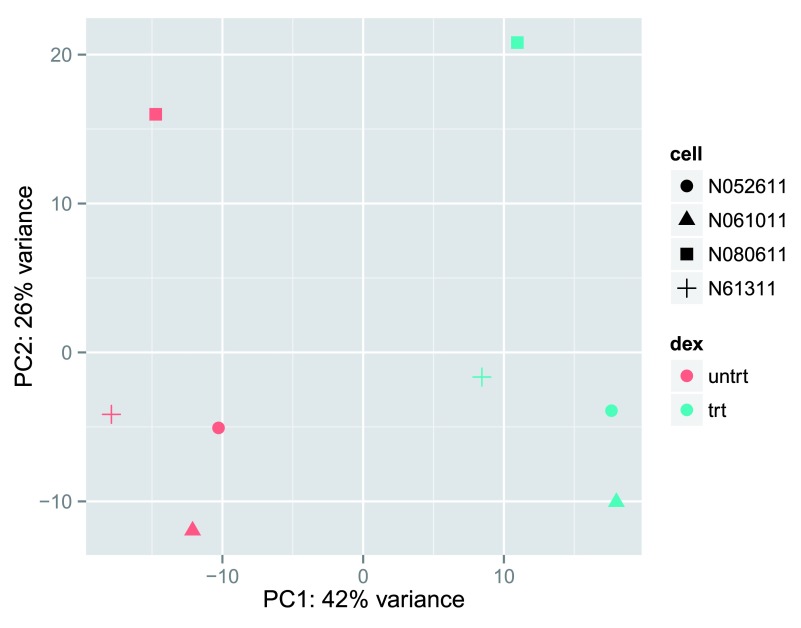
PCA plot using the rlog-transformed values with custom *ggplot2* code. Here we specify cell line (plotting symbol) and dexamethasone treatment (color).



We can then use this data to build up a second plot in Figure 6, specifying that the color of the points should reflect dexamethasone treatment and the shape should reflect the cell line.

                        library
                        (
                        "ggplot2"
                        )
                    




                        ggplot
                        (data, 
                        aes
                        (PC1, PC2, 
                        color=
                        dex, 
                        shape=
                        cell)) +   
                        geom_point
                        (
                        size=
                        3
                        ) +
   
                        xlab
                        (
                        paste0
                        (
                        "PC1: “
                        ,percentVar[
                        1
                        ],
                        “% variance"
                        )) +
   
                        ylab
                        (
                        paste0
                        (
                        "PC2: ”
                        ,percentVar[
                        2
                        ],
                        “% variance"
                        ))
                    


From the PCA plot, we see that the differences between cells (the different plotting shapes) are considerable, though not stronger than the differences due to treatment with dexamethasone (red vs blue color). This shows why it will be important to account for this in differential testing by using a paired design (“paired”, because each dex treated sample is paired with one untreated sample from the *same* cell line). We are already set up for this design by assigning the formula ~ cell + dex earlier.

### MDS plot

**Figure 7.**
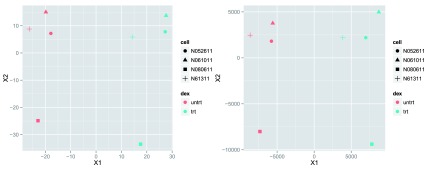
MDS plots. Shown are the plots based on the rlog-transformed values (left) and the *Poisson Distance* (right).



Another plot, very similar to the PCA plot, can be made using the *multidimensional scaling* (MDS) function in base R. This is useful when we don’t have a matrix of data, but only a matrix of distances. Here we compute the MDS for the distances calculated from the *rlog* transformed counts and plot these ( Figure 7):

                        mdsData <- 
                        data.frame
                        (
                        cmdscale
                        (sampleDistMatrix))

                        mds <- 
                        cbind
                        (mdsData, 
                        as.data.frame
                        (
                        colData
                        (rld)))

                        ggplot
                        (mds, 
                        aes
                        (X1,X2,
                        color=
                        dex,
                        shape=
                        cell)) + 
                        geom_point
                        (
                        size=
                        3
                        )
                    


Creating the same plot for the *PoissonDistance* (also Figure 7):



                        mdsPoisData <- 
                        data.frame
                        (
                        cmdscale
                        (samplePoisDistMatrix))

                        mdsPois <- 
                        cbind
                        (mdsPoisData, 
                        as.data.frame
                        (
                        colData
                        (dds)))

                        ggplot
                        (mdsPois, 
                        aes
                        (X1,X2,
                        color=
                        dex,
                        shape=
                        cell)) + 
                        geom_point
                        (
                        size=
                        3
                        )
                    


## Differential expression analysis

### Running the differential expression pipeline

As we have already specified an experimental design when we created the *DESeqDataSet*, we can run the differential expression pipeline on the raw counts with a single call to the function *DESeq*:



                        dds <- 
                        DESeq
                        (dds)
                    


This function will print out a message for the various steps it performs. These are described in more detail in the manual page for *DESeq*, which can be accessed by typing ?DESeq. Briefly these are: the estimation of size factors (controlling for differences in the sequencing depth of the samples), the estimation of dispersion values for each gene, and fitting a generalized linear model.

A *DESeqDataSet* is returned that contains all the fitted parameters within it, and the following section describes how to extract out results tables of interest from this object.

### Building the results table

Calling *results* without any arguments will extract the estimated log2 fold changes and *p* values for the last variable in the design formula. If there are more than 2 levels for this variable, *results* will extract the results table for a comparison of the last level over the first level. This comparison is printed at the top of the output: dex trt vs untrt.



                        (res <- 
                        results
                        (dds))
                    




                        ## log2 fold change (MAP): dex trt vs untrt
## Wald test p-value: dex trt vs untrt
## DataFrame with 29391 rows and 6 columns
##		      baseMean log2FoldChange	   lfcSE       stat	pvalue	       padj
##		     <numeric>	    <numeric>  <numeric>  <numeric>   <numeric>   <numeric>
## ENSG00000000003 708.6021697	  -0.37423028 0.09872592 -3.7905980 0.000150285 0.001224146
## ENSG00000000419 520.2979006	   0.20214241 0.10929202  1.8495625 0.064376631 0.189223539
## ENSG00000000457 237.1630368	   0.03624420 0.13682871  0.2648874 0.791096181 0.907794192
## ENSG00000000460  57.9326331	  -0.08520813 0.24645454 -0.3457357 0.729541350 0.875201476
## ENSG00000000938   0.3180984	  -0.11522629 0.14589383 -0.7897955 0.429647219		 NA
## ...			   ...		  ...	     ...	...	    ...		...
## ENSG00000273485   1.2864477	   0.03490688  0.2986168  0.1168952   0.9069431		 NA
## ENSG00000273486  15.4525365	  -0.09662406  0.3385222 -0.2854290   0.7753155   0.8990371
## ENSG00000273487   8.1632350	   0.56255493  0.3731295  1.5076666   0.1316399   0.3177048
## ENSG00000273488   8.5844790	   0.10794134  0.3680474  0.2932811   0.7693073   0.8960855
## ENSG00000273489   0.2758994	   0.11249632  0.1420250  0.7920882   0.4283092		 NA
                    


As res is a *DataFrame* object, it carries metadata with information on the meaning of the columns:



                        mcols
                        (res, 
                        use.names=
                        TRUE
                        )
                    




                        ## DataFrame with 6 rows and 2 columns
##			     type				description
##		      <character>			        <character>
## baseMean          intermediate mean of normalized counts for all samples
## log2FoldChange         results  log2 fold change (MAP): dex trt vs untrt
## lfcSE		  results          standard error: dex trt vs untrt
## stat			  results          Wald statistic: dex trt vs untrt
## pvalue		  results       Wald test p-value: dex trt vs untrt
## padj			  results		       BH adjusted p-values
                    


The first column, baseMean, is a just the average of the normalized count values, dividing by size factors, taken over all samples in the *DESeqDataSet*. The remaining four columns refer to a specific contrast, namely the comparison of the trt level over the untrt level for the factor variable dex. We will find out below how to obtain other contrasts.

The column log2FoldChange is the effect size estimate. It tells us how much the gene’s expression seems to have changed due to treatment with dexamethasone in comparison to untreated samples. This value is reported on a logarithmic scale to base 2: for example, a log2 fold change of 1.5 means that the gene’s expression is increased by a multiplicative factor of 2 ^1.5^ ≈ 2.82.

Of course, this estimate has an uncertainty associated with it, which is available in the column lfcSE, the standard error estimate for the log2 fold change estimate. We can also express the uncertainty of a particular effect size estimate as the result of a statistical test. The purpose of a test for differential expression is to test whether the data provides sufficient evidence to conclude that this value is really different from zero. *DESeq2* performs for each gene a *hypothesis test* to see whether evidence is sufficient to decide against the *null hypothesis* that there is zero effect of the treatment on the gene and that the observed difference between treatment and control was merely caused by experimental variability (i.e., the type of variability that you can expect between different samples in the same treatment group). As usual in statistics, the result of this test is reported as a *p* value, and it is found in the column pvalue. Remember that a *p* value indicates the probability that a fold change as strong as the observed one, or even stronger, would be seen under the situation described by the null hypothesis.

We can also summarize the results with the following line of code, which reports some additional information, that will be covered in later sections.



                        summary
                        (res)
                    




                        ##
## out of 29391 with nonzero total read count
## adjusted p-value < 0.1
## LFC > 0 (up)     : 2647, 9%
## LFC < 0 (down)   : 2250, 7.7%
## outliers [1]     : 0, 0%
## low counts [2]   : 11756, 40%
## (mean count < 5.2)
## [1] see 'cooksCutoff' argument of ?results
## [2] see 'independentFiltering' argument of ?results
                    


Note that there are many genes with differential expression due to dexamethasone treatment at the FDR level of 10%. This makes sense, as the smooth muscle cells of the airway are known to react to glucocorticoid steroids. However, there are two ways to be more strict about which set of genes are considered significant:

- lower the false discovery rate threshold (the threshold on padj in the results table)
- raise the log2 fold change threshold from 0 using the lfcThreshold argument of *results*

If we lower the false discovery rate threshold, we should also tell this value to results(), so that the function will use an alternative threshold for the optimal independent filtering step:



                        res
                        .05 
                        <- 
                        results
                        (dds, 
                        alpha=
                        .05
                        )

                        table
                        (res
                        .05
                        $padj < 
                        .05
                        )
                    




                        ##
## FALSE  TRUE
## 12095  4070
                    


If we want to raise the log2 fold change threshold, so that we test for genes that show more substantial changes due to treatment, we simply supply a value on the log2 scale. For example, by specifying lfcThreshold=1, we test for genes that show significant effects of treatment on gene counts more than doubling or less than halving, because 2 ^1^ = 2.



                        resLFC1 <- 
                        results
                        (dds, 
                        lfcThreshold=
                        1
                        )

                        table
                        (resLFC1$padj < 
                        0.1
                        )
                    




                        ##
## FALSE  TRUE
## 14492   204
                    


Sometimes a subset of the *p* values in res will be NA (“not available”). This is *DESeq*’s way of reporting that all counts for this gene were zero, and hence no test was applied. In addition, *p* values can be assigned NA if the gene was excluded from analysis because it contained an extreme count outlier. For more information, see the outlier detection section of the *DESeq2* vignette.

If you use the results from an R analysis package in published research, you can find the proper citation for the software by typing citation("pkgName"), where you would substitute the name of the package for pkgName. Citing methods papers helps to support and reward the individuals who put time into open source software for genomic data analysis.

### Other comparisons

In general, the results for a comparison of any two levels of a variable can be extracted using the contrast argument to *results*. The user should specify three values: the name of the variable, the name of the level for the numerator, and the name of the level for the denominator. Here we extract results for the log2 of the fold change of one cell line over another:



                        results
                        (dds, 
                        contrast=c
                        (
                        "cell"
                        , 
                        "N061011"
                        , 
                        "N61311"
                        ))
                    




                        ## log2 fold change (MAP): cell N061011 vs N61311
## Wald test p-value: cell N061011 vs N61311
## DataFrame with 29391 rows and 6 columns
##		      baseMean log2FoldChange	   lfcSE         stat	 pvalue	     padj
##		     <numeric>	    <numeric>  <numeric>    <numeric> <numeric> <numeric>
## ENSG00000000003 708.6021697	   0.29054171 0.13600021   2.13633281 0.0326523 0.1981039
## ENSG00000000419 520.2979006	  -0.05069310 0.14916364  -0.33984894 0.7339703 0.9238903
## ENSG00000000457 237.1630368	   0.01474318 0.18161982   0.08117606 0.9353019 0.9862379
## ENSG00000000460  57.9326331	   0.20241839 0.28064506   0.72126120 0.4707488 0.8108444
## ENSG00000000938   0.3180984	   0.00000000 0.07169692   0.00000000 1.0000000	       NA
## ...			   ...		  ...	     ...	...	    ...	      ...
## ENSG00000273485   1.2864477	 -0.180248108 0.16456445 -1.095304052 0.2733835	       NA
## ENSG00000273486  15.4525365   -0.029979349 0.30827915 -0.097247409 0.9225299	       NA
## ENSG00000273487   8.1632350	 -0.001914497 0.28117903 -0.006808819 0.9945674	       NA
## ENSG00000273488   8.5844790	  0.380608540 0.29209485  1.303030638 0.1925643	       NA
## ENSG00000273489   0.2758994	  0.000000000 0.06955643  0.000000000 1.0000000	       NA
                    


If results for an interaction term are desired, the name argument of *results* should be used. Please see the help for the *results* function for more details.

### Multiple testing

In high-throughput biology, we are careful to not use the *p* values directly as evidence against the null, but to correct for *multiple testing*. What would happen if we were to simply threshold the *p* values at a low value, say 0.05? There are 5722 genes with a *p* value below 0.05 among the 29391 genes, for which the test succeeded in reporting a *p* value:



                        sum
                        (res$pvalue < 
                        0.05
                        , 
                        na.rm=
                        TRUE
                        )
                    




                        ## [1] 5722
                    




                        sum
                        (
                        !is.na
                        (res$pvalue))
                    




                        ## [1] 29391
                    


Now, assume for a moment that the null hypothesis is true for all genes, i.e., no gene is affected by the treatment with dexamethasone. Then, by the definition of the *p* value, we expect up to 5% of the genes to have a *p* value below 0.05. This amounts to 1470 genes. If we just considered the list of genes with a *p* value below 0.05 as differentially expressed, this list should therefore be expected to contain up to 1470/5722 = 26% false positives.

*DESeq2* uses the Benjamini-Hochberg (BH) adjustment ^20^ as implemented in the base R *p.adjust* function; in brief, this method calculates for each gene an adjusted *p* value that answers the following question: if one called significant all genes with an adjusted *p* value less than or equal to this gene’s adjusted *p* value threshold, what would be the fraction of false positives (the *false discovery rate*, FDR) among them, in the sense of the calculation outlined above? These values, called the BH-adjusted *p* values, are given in the column padj of the res object.

The FDR is a useful statistic for many high-throughput experiments, as we are often interested in reporting or focusing on a set of interesting genes, and we would like to put an upper bound on the percent of false positives in this set.

Hence, if we consider a fraction of 10% false positives acceptable, we can consider all genes with an adjusted *p* value below 10% = 0.1 as significant. How many such genes are there?



                        sum
                        (res$padj < 
                        0.1
                        , 
                        na.rm=
                        TRUE
                        )
                    




                        ## [1] 4897
                    


We subset the results table to these genes and then sort it by the log2 fold change estimate to get the significant genes with the strongest down-regulation:



                        resSig <- 
                        subset
                        (res, padj < 
                        0.1
                        )

                        head
                        (resSig[ 
                        order
                        (resSig$log2FoldChange), ])
                    




                        ## log2 fold change (MAP): dex trt vs untrt
## Wald test p-value: dex trt vs untrt
## DataFrame with 6 rows and 6 columns
##		    baseMean log2FoldChange	lfcSE       stat       pvalue	      padj
##		   <numeric>	  <numeric> <numeric>  <numeric>    <numeric>    <numeric>
## ENSG00000162692 508.17023	  -3.452454 0.1763751 -19.574503 2.551125e-85 3.460700e-82
## ENSG00000146006  46.80760	  -2.856273 0.3366877  -8.483451 2.186122e-17 1.073879e-15
## ENSG00000105989 333.21469	  -2.850960 0.1754638 -16.248133 2.302720e-59 1.194366e-56
## ENSG00000214814 243.27698	  -2.759539 0.2224907 -12.402938 2.519140e-35 4.113429e-33
## ENSG00000267339  26.23357	  -2.743928 0.3511985  -7.813041 5.582443e-15 2.182846e-13
## ENSG00000013293 244.49733	  -2.646116 0.1981216 -13.356020 1.092517e-40 2.240295e-38
                    


... and with the strongest up-regulation:



                        head
                        (resSig[ 
                        order
                        (resSig$log2FoldChange, 
                        decreasing=
                        TRUE
                        ), ])
                    




                        ## log2 fold change (MAP): dex trt vs untrt
## Wald test p-value: dex trt vs untrt
## DataFrame with 6 rows and 6 columns
##		    baseMean log2FoldChange	lfcSE       stat        pvalue	       padj
##		   <numeric>	  <numeric> <numeric>  <numeric>     <numeric>     <numeric>
## ENSG00000179593  67.24305	   4.880507 0.3308119   14.75312  2.937594e-49  9.418996e-47
## ENSG00000109906 385.07103	   4.860877 0.3321627   14.63403  1.704000e-48  5.181040e-46
## ENSG00000152583 997.43977	   4.315374 0.1723805   25.03400 2.608143e-138 4.599460e-134
## ENSG00000250978  56.31819	   4.090157 0.3288246   12.43872  1.610666e-35  2.679631e-33
## ENSG00000163884 561.10717	   4.078073 0.2103212   19.38974  9.421379e-84  1.038413e-80
## ENSG00000168309 159.52692	   3.991146 0.2547755   15.66534  2.610147e-55  1.180255e-52
                    


## Plotting results

**Figure 8.**
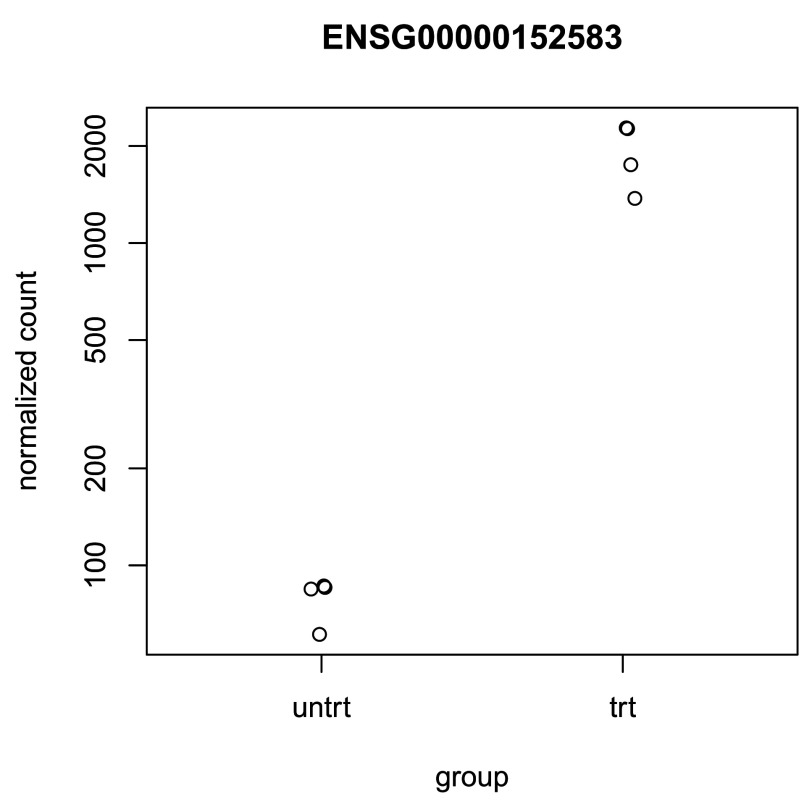
Normalized counts for a single gene over treatment group.



A quick way to visualize the counts for a particular gene is to use the *plotCounts* function that takes as arguments the *DESeqDataSet*, a gene name, and the group over which to plot the counts ( Figure 8).

                    topGene <- 
                    rownames
                    (res)[
                    which.min
                    (res$padj)]

                    plotCounts
                    (dds, 
                    gene=
                    topGene, 
                    intgroup=c
                    (
                    "dex"
                    ))
                


**Figure 9.**
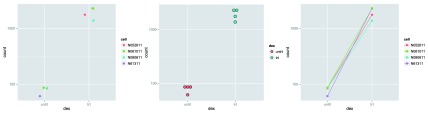
Normalized counts over treatment using different *ggplot2* styles. The plots are customized using *ggplot2* options for jitter (left), dots (middle), or with lines connecting cell line (right). Note that the *DESeq2* test that was used takes into account the cell line effect, so the rightmost figure more closely depicts the difference being tested.



We can also make custom plots using the *ggplot* function from the *ggplot2* package ( Figure 9).

                    data <- 
                    plotCounts
                    (dds, 
                    gene=
                    topGene, 
                    intgroup=c
                    (
                    “dex"
                    ,
                    “cell"
                    ), 
                    returnData=
                    TRUE
                    )

                    ggplot
                    (data, 
                    aes
                    (
                    x=
                    dex, 
                    y=
                    count, 
                    color=
                    cell)) +
  
                    scale_y_log10
                    () +
  
                    geom_point
                    (
                    position=position_jitter
                    (
                    width=
                    .1
                    ,
                    height=
                    0
                    ), 
                    size=
                    3
                    )
                




                    ggplot
                    (data, 
                    aes
                    (
                    x=
                    dex, 
                    y=
                    count, 
                    fill=
                    dex)) +
  
                    scale_y_log10
                    () +
  
                    geom_dotplot
                    (
                    binaxis=
                    “y"
                    , 
                    stackdir=
                    “center"
                    )
                




                    ggplot
                    (data, 
                    aes(
                    x=
                    dex, 
                    y=
                    count, 
                    color=
                    cell, 
                    group=
                    cell)) +
  
                    scale_y_log10
                    () + 
                    geom_point
                    (
                    size=
                    3
                    ) + 
                    geom_line
                    ()
                


**Figure 10.**
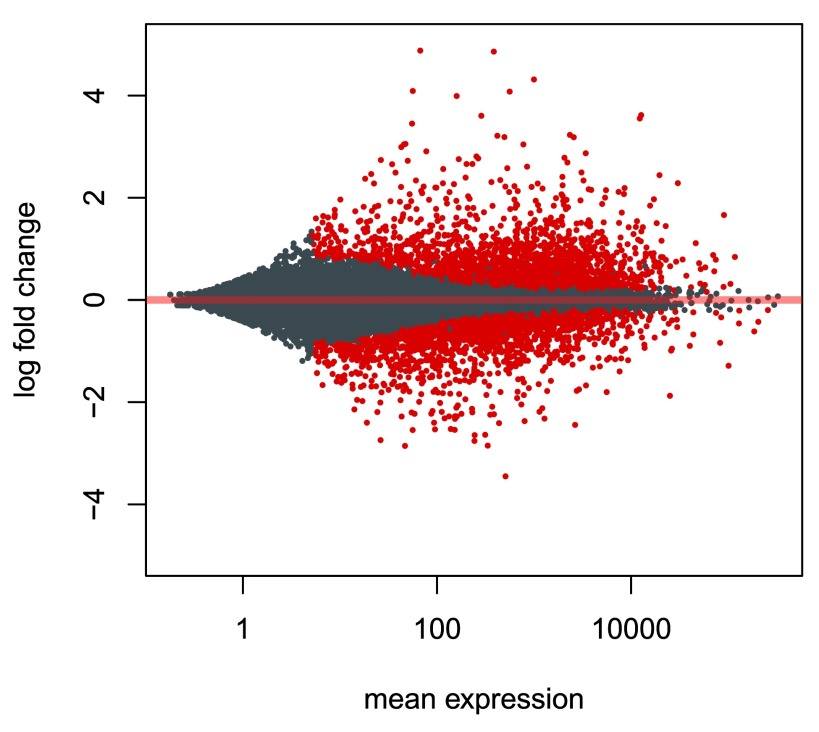
An MA-plot of changes induced by treatment. The log2 fold change for a particular comparison is plotted on the y-axis and the average of the counts normalized by size factor is shown on the x-axis (“M” for minus, because a log ratio is equal to log minus log, and “A” for average). Each gene is represented with a dot. Genes with an adjusted *p* value below a threshold (here 0.1, the default) are shown in red.



An *MA-plot* ^21^ provides a useful overview for an experiment with a two-group comparison ( Figure 10).

                    plotMA
                    (res, 
                    ylim=c
                    (-
                    5
                    ,
                    5
                    ))
                


The *DESeq2* package uses statistical techniques to moderate log2 fold changes from genes with very low counts and highly variable counts, as can be seen by the narrowing of the vertical spread of points on the left side of the MA-plot. For a detailed explanation of the rationale of moderated fold changes, please see the *DESeq2* paper ^2^. This plot demonstrates that only genes with a large average normalized count contain sufficient information to yield a significant call.

**Figure 11.**
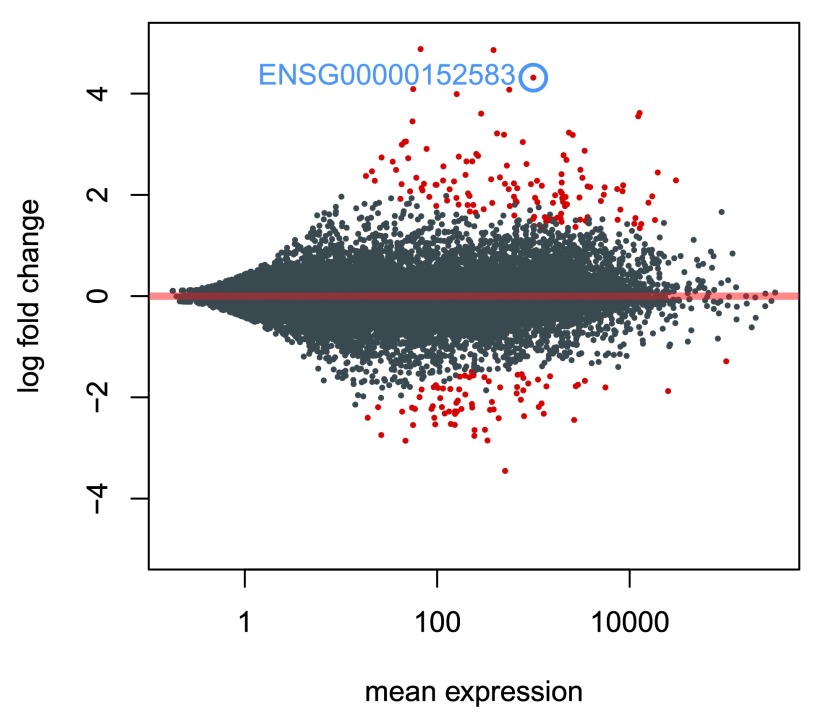
An MA-plot of a test for large log2 fold changes. The red points indicate genes for which the log2 fold change was significantly higher than 1 or less than -1 (treatment resulting in more than doubling or less than halving of the normalized counts) with adjusted *p* value less than 0.1. The point circled in blue indicates the gene with the lowest adjusted *p* value.



We can also make an MA-plot for the results table in which we raised the log2 fold change threshold ( Figure 11). We can label individual points on the MA-plot as well. Here we use the *with* R function to plot a circle and text for a selected row of the results object. Within the *with* function, only the baseMean and log2FoldChange values for the selected rows of res are used.

                    plotMA
                    (resLFC1, 
                    ylim=c
                    (-
                    5
                    ,
                    5
                    ))
topGene <- 
                    rownames
                    (resLFC1)[
                    which.min
                    (resLFC1$padj)]

                    with
                    (resLFC1[topGene, ], {
  
                    points
                    (baseMean, log2FoldChange, 
                    col=
                    “dodgerblue"
                    , 
                    cex=
                    2
                    , 
                    lwd=
                    2
                    )
  
                    text
                    (baseMean, log2FoldChange, topGene, 
                    pos=
                    2
                    , 
                    col=
                    “dodgerblue"
                    )
})
                


**Figure 12.**
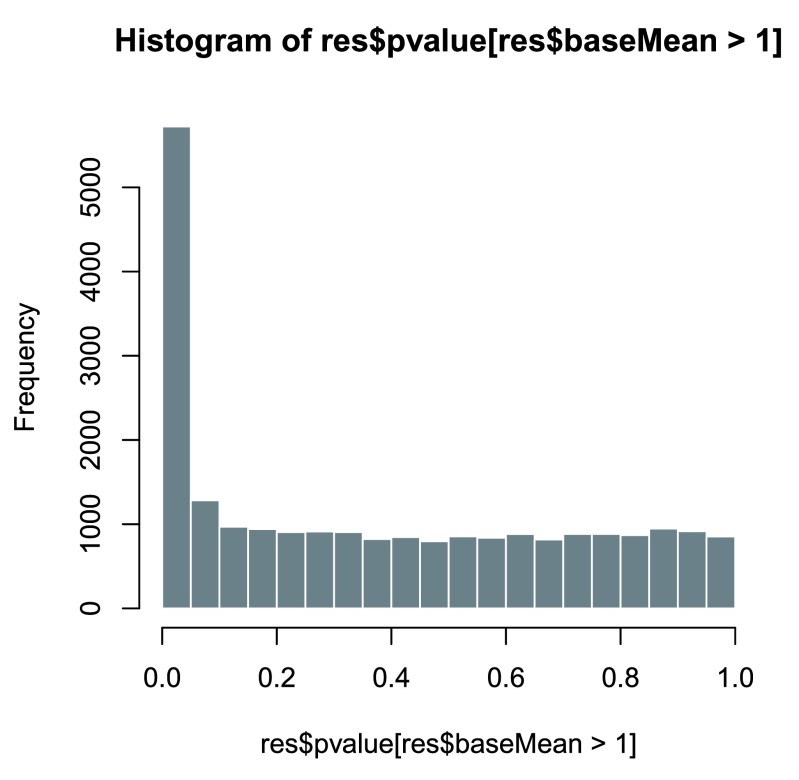
Histogram of *p* values for genes with mean normalized count larger than 1.



Another useful diagnostic plot is the histogram of the *p* values ( Figure 12). This plot is best formed by excluding genes with very small counts, which otherwise generate spikes in the histogram.

                    hist
                    (res$pvalue[res$baseMean > 
                    1
                    ], 
                    breaks=
                    0
                    :
                    20
                    /
                    20
                    , 
                    col=
                    “grey50"
                    , 
                    border=
                    “white"
                    )
                


### Gene clustering

In the sample distance heatmap made previously, the dendrogram at the side shows us a hierarchical clustering of the samples. Such a clustering can also be performed for the genes. Since the clustering is only relevant for genes that actually carry a signal, one usually would only cluster a subset of the most highly variable genes. Here, for demonstration, let us select the 20 genes with the highest variance across samples. We will work with the *rlog* transformed counts:



                        library
                        (
                        "genefilter"
                        )
topVarGenes <- 
                        head
                        (
                        order
                        (
                        rowVars
                        (
                        assay
                        (rld)),
                        decreasing=
                        TRUE
                        ),
                        20
                        )
                    


**Figure 13.**
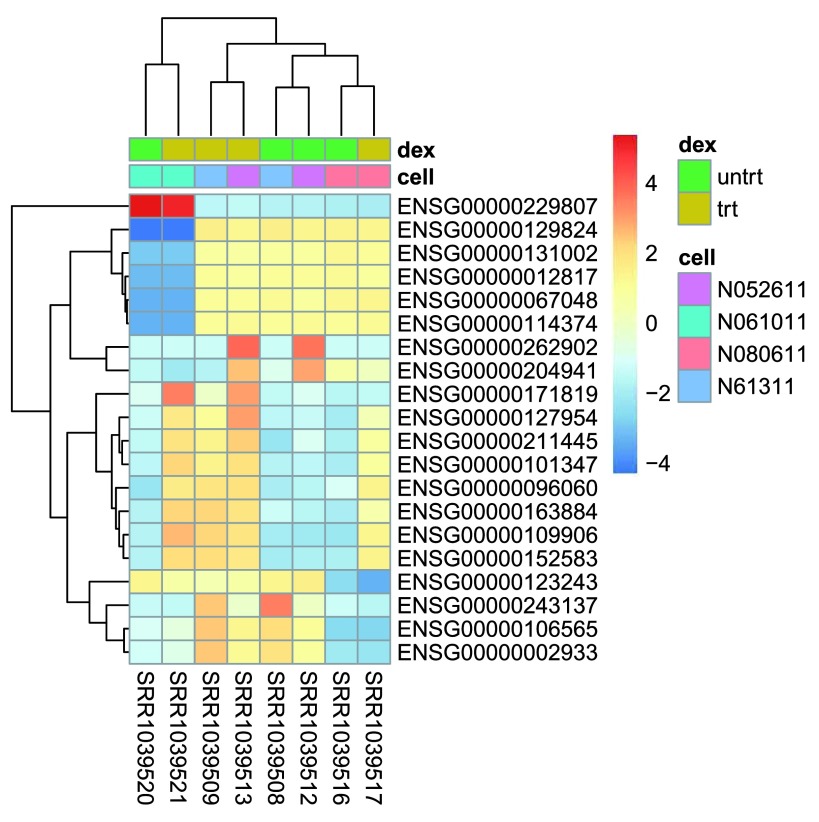
Heatmap of relative rlog-transformed values across samples. Treatment status and cell line information are shown with colored bars at the top of the heatmap. Note that a set of genes at the top of the heatmap are separating the N061011 cell line from the others. In the center of the heatmap, we see a set of genes for which the dexamethasone treated samples have higher gene expression.



The heatmap becomes more interesting if we do not look at absolute expression strength but rather at the amount by which each gene deviates in a specific sample from the gene’s average across all samples. Hence, we center each genes’ values across samples, and plot a heatmap ( Figure 13). We provide a *data.frame* that instructs the *pheatmap* function how to label the columns.

                        mat <- 
                        assay
                        (rld)[ topVarGenes, ]
mat <- mat - 
                        rowMeans
                        (mat)
df <- 
                        as.data.frame
                        (
                        colData
                        (rld)[,
                        c
                        (
                        “cell"
                        ,
                        “dex"
                        )])

                        pheatmap
                        (mat, 
                        annotation_col=
                        df)
                    


### Independent filtering

The MA plot highlights an important property of RNA-seq data. For weakly expressed genes, we have no chance of seeing differential expression, because the low read counts suffer from such high Poisson noise that any biological effect is drowned in the uncertainties from the sampling at a low rate. We can also show this by examining the ratio of small *p* values (say, less than, 0.05) for genes binned by mean normalized count. We will use the results table subjected to the threshold to show what this looks like in a case when there are few tests with small *p* value.

**Figure 14.**
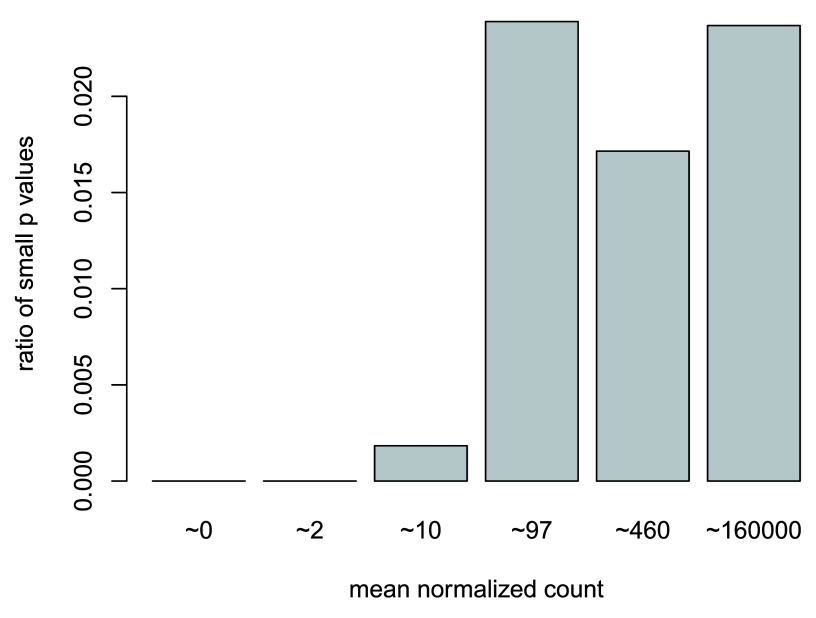
The ratio of small *p* values for genes binned by mean normalized count. Here the *p* values are for a test of log2 fold change greater than 1 or less than -1. This plot demonstrates that genes with low mean count are underpowered, and best excluded before multiple test correction.



In the following code chunk, we create bins using the *quantile* function, bin the genes by base mean using *cut*, rename the levels of the bins using the middle point, calculate the ratio of *p* values less than 0.05 for each bin, and finally plot these ratios ( Figure 14).

                        qs <- 
                        c
                        (
                        0
                        , 
                        quantile
                        (resLFC1$baseMean[resLFC1$baseMean > 
                        0
                        ], 
                        0
                        :
                        6
                        /
                        6
                        ))
bins <- 
                        cut
                        (resLFC1$baseMean, qs) 

                        levels
                        (bins) <- 
                        paste0
                        (
                        "~"
                        ,
                        round
                        (
                        signif
                        (
                        .5
                        *qs[
                        -1
                        ] + 
                        .5
                        *qs[
                        -length
                        (qs)],
                        2
                        )))
ratios <- 
                        tapply
                        (resLFC1$pvalue, bins, function(p) 
                        mean
                        (p < 
                        .05
                        , 
                        na.rm=
                        TRUE
                        ))

                        barplot
                        (ratios, 
                        xlab=
                        “mean normalized count"
                        , 
                        ylab=
                        “ratio of small p values"
                        )
                    


At first sight, there may seem to be little benefit in filtering out these genes. After all, the test found them to be non-significant anyway. However, these genes have an influence on the multiple testing adjustment, whose performance improves if such genes are removed. By removing the low count genes from the input to the FDR procedure, we can find more genes to be significant among those that we keep, and so improved the power of our test. This approach is known as *independent filtering*.

The *DESeq2* software automatically performs independent filtering that maximizes the number of genes with adjusted *p* value less than a critical value (by default, alpha is set to 0.1). This automatic independent filtering is performed by, and can be controlled by, the *results* function.

The term *independent* highlights an important caveat. Such filtering is permissible only if the statistic that we filter on (here the mean of normalized counts across all samples) is independent of the actual test statistic (the *p* value) under the null hypothesis. Otherwise, the filtering would invalidate the test and consequently the assumptions of the BH procedure. The independent filtering software used inside *DESeq2* comes from the *genefilter* package, that contains a reference to a paper describing the statistical foundation for independent filtering ^22^.

## Annotating and exporting results

Our result table so far only contains information about Ensembl gene IDs, but alternative gene names may be more informative for collaborators. Bioconductor’s annotation packages help with mapping various ID schemes to each other. We load the *AnnotationDbi* package and the annotation package *org.Hs.eg.db*:



                    library
                    (
                    "AnnotationDbi"
                    )

                    library
                    (
                    "org.Hs.eg.db"
                    )
                


This is the organism annotation package (“org”) for *Homo sapiens* (“Hs”), organized as an *AnnotationDbi* database package (“db”), using Entrez Gene IDs (“eg”) as primary key. To get a list of all available key types, use:



                    columns
                    (org.Hs.eg.db)
                




                    ##  [1] "ENTREZID"     "PFAM"	      "IPI"	     "PROSITE"	  "ACCNUM"	 "ALIAS"
##  [7] "CHR"	       "CHRLOC"	      "CHRLOCEND"    "ENZYME"	  "MAP"		 "PATH"
## [13] "PMID"	       "REFSEQ"	      "SYMBOL"	     "UNIGENE"	  "ENSEMBL"	 "ENSEMBLPROT"
## [19] "ENSEMBLTRANS" "GENENAME"     "UNIPROT"	     "GO"	  "EVIDENCE"	 "ONTOLOGY"
## [25] "GOALL"	       "EVIDENCEALL"  "ONTOLOGYALL"  "OMIM"	  "UCSCKG"
                


We can use the *mapIds* function to add individual columns to our results table. We provide the row names of our results table as a key, and specify that keytype=ENSEMBL. The column argument tells the *mapIds* function which information we want, and the multiVals argument tells the function what to do if there are multiple possible values for a single input value. Here we ask to just give us back the first one that occurs in the database. To add the gene symbol and Entrez ID, we call *mapIds* twice.



                    res$symbol <- 
                    mapIds
                    (org.Hs.eg.db,
		       
                    keys=row.names
                    (res),
		       
                    column=
                    "SYMBOL"
                    ,
		       
                    keytype=
                    "ENSEMBL"
                    ,
		       
                    multiVals=
                    "first"
                    )
res$entrez <- 
                    mapIds
                    (org.Hs.eg.db,
		       
                    keys=row.names
                    (res),
		       
                    column=
                    "ENTREZID"
                    ,
		       
                    keytype=
                    "ENSEMBL"
                    ,
		       
                    multiVals=
                    "first"
                    )
                


Now the results have the desired external gene IDs:



                    resOrdered <- res[
                    order
                    (res$padj),]

                    head
                    (resOrdered)
                




                    ## log2 fold change (MAP): dex trt vs untrt
## Wald test p-value: dex trt vs untrt
## DataFrame with 6 rows and 8 columns
##		     baseMean log2FoldChange	 lfcSE      stat	pvalue	        padj
##		    <numeric>	   <numeric> <numeric> <numeric>     <numeric>     <numeric>
## ENSG00000152583   997.4398	    4.315374 0.1723805  25.03400 2.608143e-138 4.599460e-134
## ENSG00000165995   495.0929	    3.188413 0.1277306  24.96201 1.581556e-137 1.394537e-133
## ENSG00000101347 12703.3871	    3.617791 0.1499256  24.13057 1.194421e-128 6.472441e-125
## ENSG00000120129  3409.0294	    2.871106 0.1190242  24.12204 1.468090e-128 6.472441e-125
## ENSG00000189221  2341.7673	    3.230290 0.1373499  23.51869 2.626434e-122 9.263434e-119
## ENSG00000211445 12285.6151	    3.552498 0.1589749  22.34628 1.312430e-110 3.857449e-107
##		        symbol      entrez
##	           <character> <character>
## ENSG00000152583     SPARCL1        8404
## ENSG00000165995      CACNB2         783
## ENSG00000101347      SAMHD1       25939
## ENSG00000120129       DUSP1        1843
## ENSG00000189221        MAOA        4128
## ENSG00000211445        GPX3        2878
                


### Exporting results

You can easily save the results table in a CSV file, that you can then share or load with a spreadsheet program such as Excel. The call to *as.data.frame* is necessary to convert the *DataFrame* object ( *IRanges* package) to a *data.frame* object that can be processed by *write.csv*. Here, we take just the top 100 genes for demonstration.



                        resOrderedDF <- 
                        as.data.frame
                        (resOrdered)[
                        1
                        :
                        100
                        ,]

                        write.csv
                        (resOrderedDF, 
                        file=
                        “results.csv"
                        )
                    


Another more sophisticated package for exporting results from various Bioconductor analysis packages is the *ReportingTools* package ^23^. *ReportingTools* will automatically generate dynamic HTML documents, including links to external databases using gene identifiers and boxplots summarizing the normalized counts across groups. See the *ReportingTools* vignettes for full details. The simplest version of creating a dynamic *ReportingTools* report is performed with the following code:



                        library
                        (
                        "ReportingTools"
                        )
htmlRep <- 
                        HTMLReport
                        (
                        shortName=
                        “report"
                        , 
                        title=
                        "My report"
                        ,
		        
                        reportDirectory=
                        "./report"
                        )

                        publish
                        (resOrderedDF, htmlRep)
url <- 
                        finish
                        (htmlRep)

                        browseURL
                        (url)
                    


### Plotting fold changes in genomic space

If we have used the *summarizeOverlaps* function to count the reads, then our *DESeqDataSet* object is built on top of ready-to-use Bioconductor objects specifying the genomic ranges of the genes. We can therefore easily plot our differential expression results in genomic space. While the *results* function by default returns a *DataFrame*, using the format argument, we can ask for *GRanges* or *GRangesList* output.



                        (resGR <- 
                        results
                        (dds, 
                        lfcThreshold=1
                        , 
                        format=
                        “GRanges"
                        ))
                    




                        ## GRanges object with 29391 ranges and 6 metadata columns:
##		     seqnames		      ranges strand   |		 baseMean      log2FoldChange
##		        <Rle>		   <IRanges>  <Rle>   |		<numeric>	    <numeric>
##   ENSG00000000003	    X [ 99883667,  99894988]	  -   |  708.602169691234  -0.374230275713608
##   ENSG00000000419	   20 [ 49551404,  49575092]	  -   |  520.297900552084   0.202142414893829
##   ENSG00000000457	    1 [169818772, 169863408]	  -   |  237.163036796015  0.0362442044069634
##   ENSG00000000460	    1 [169631245, 169823221]	  +   |  57.9326331250967 -0.0852081341501509
##   ENSG00000000938	    1 [ 27938575,  27961788]	  -   | 0.318098378392895  -0.115226286496983
##   		 ...	  ...			 ...	... ...	     	      ...		  ...
##   ENSG00000273485	   10 [105209953, 105210609]	  +   |	 1.28644765243289  0.0349068755370733
##   ENSG00000273486	    3 [136556180, 136557863]	  -   |	 15.4525365439045 -0.0966240584195589
##   ENSG00000273487	    1 [ 92654794,  92656264]	  +   |	  8.1632349843654   0.562554926745931
##   ENSG00000273488	    3 [100080031, 100080481]	  +   |	 8.58447903624707   0.107941339873098
##   ENSG00000273489	    7 [131178723, 131182453]	  -   | 0.275899382507797   0.112496317318932
##				 lfcSE	    stat    pvalue      padj

##			     <numeric> <numeric> <numeric> <numeric>
##   ENSG00000000003 0.098725921418789	       0	 1	   1
##   ENSG00000000419 0.109292016112333	       0	 1	   1
##   ENSG00000000457 0.136828705643854	       0	 1	   1
##   ENSG00000000460 0.246454541134745	       0	 1	   1
##   ENSG00000000938 0.145893828687766	       0	 1	<NA>
##  		 ...		   ...	     ...       ...	 ...
##   ENSG00000273485 0.298616754374375	       0	 1	<NA>
##   ENSG00000273486 0.338522172238827	       0	 1	<NA>
##   ENSG00000273487 0.373129529007307	       0	 1	<NA>
##   ENSG00000273488 0.368047431494871	       0	 1	<NA>
##   ENSG00000273489 0.142024983011115	       0	 1	<NA>
##   -------				
##   seqinfo: 722 sequences (1 circular) from an unspecified genome
                    


We need to add the symbol again for labeling the genes on the plot:



                        resGR$symbol <- 
                        mapIds
                        (org.Hs.eg.db, 
                        names
                        (resGR), 
                        "SYMBOL"
                        , 
                        "ENSEMBL"
                        )
                    


We will use the *Gviz* package for plotting the GRanges and associated metadata: the log fold changes due to dexamethasone treatment.



                        library
                        (
                        "Gviz"
                        )
                    


The following code chunk specifies a window of 1 million base pairs upstream and downstream from the gene with the smallest *p* value. We create a subset of our full results, for genes within the window We add the gene symbol as a name, if the symbol exists or is not duplicated in our subset.



                        window <- resGR[topGene] + 
                        1e6

                        strand
                        (window) <- 
                        "*"

                        resGRsub <- resGR[resGR %over% window]
naOrDup <- 
                        is.na
                        (resGRsub$symbol) | 
                        duplicated
                        (resGRsub$symbol)
resGRsub$group <- 
                        ifelse
                        (naOrDup, 
                        names
                        (resGRsub), resGRsub$symbol)
                    


We create a vector specifying if the genes in this subset had a low false discovery rate.



                        sig <- 
                        factor
                        (
                        ifelse
                        (resGRsub$padj < 
                        .1 
                        & 
                        !is.na
                        (resGRsub$padj),
                        “sig"
                        ,
                        “notsig"
                        ))
                    


**Figure 15.**
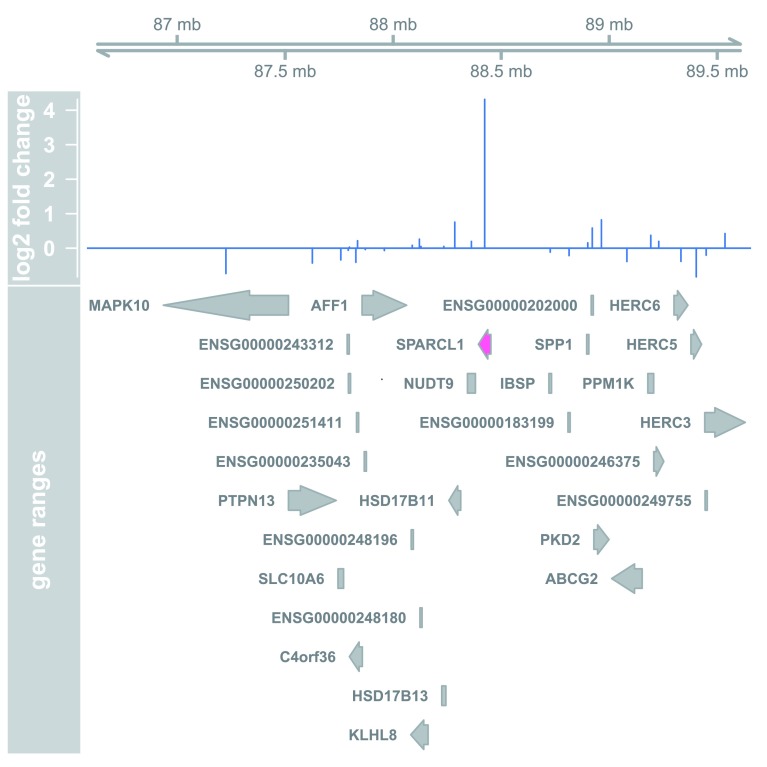
Plotting log2 fold changes in a genomic region surrounding the gene with smallest adjusted *p* value. Genes highlighted in pink have adjusted *p* value less than 0.1.



We can then plot the results using *Gviz* functions ( Figure 15). We create an axis track specifying our location in the genome, a track that will show the genes and their names, colored by significance, and a data track that will draw vertical bars showing the moderated log fold change produced by *DESeq2*, which we know are only large when the effect is well supported by the information in the counts.

                        options
                        (
                        ucscChromosomeNames=
                        FALSE
                        )
g <- 
                        GenomeAxisTrack
                        ()
a <- 
                        AnnotationTrack
                        (resGRsub, 
                        name=
                        “gene ranges"
                        , 
                        feature=
                        sig)
d <- 
                        DataTrack
                        (resGRsub, 
                        data=
                        “log2FoldChange"
                        , 
                        baseline=
                        0
                        , 
                        type=
                        “h"
                        , 
                        name=
                        “log2 fold change"
                        , 
                        strand=
                        “+"
                        )

                        plotTracks(
                        list
                        (g,d,a), 
                        groupAnnotation=
                        “group"
                        , 
                        notsig=
                        “grey"
                        , 
                        sig=
                        “hotpink"
                        )
                    


### Removing hidden batch effects

Suppose we did not know that there were different cell lines involved in the experiment, only that there was treatment with dexamethasone. The cell line effect on the counts then would represent some hidden and unwanted variation that might be affecting many or all of the genes in the dataset. We can use statistical methods designed for RNA-seq from the *sva* package ^24^ to detect such groupings of the samples, and then we can add these to the *DESeqDataSet* design, in order to account for them. The *SVA* package uses the term *surrogate variables* for the estimated variables that we want to account for in our analysis. Another package for detecting hidden batches is the *RUVSeq* package ^25^, with the acronym “Remove Unwanted Variation”.



                        library
                        (
                        "sva"
                        )
                    


Below we obtain a matrix of normalized counts for which the average count across samples is larger than 1. As we described above, we are trying to recover any hidden batch effects, supposing that we do not know the cell line information. So we use a full model matrix with the *dex* variable, and a reduced, or null, model matrix with only an intercept term. Finally we specify that we want to estimate 2 surrogate variables. For more information read the manual page for the *svaseq* function by typing ?svaseq.



                        dat <- 
                        counts
                        (dds, 
                        normalized=
                        TRUE
                        )
idx <- 
                        rowMeans
                        (dat) > 
                        1

                        dat <- dat[idx,]
mod <- 
                        model.matrix
                        (~ dex, 
                        colData
                        (dds))
mod0 <- 
                        model.matrix
                        (~ 
                        1
                        , 
                        colData
                        (dds))
svseq <- 
                        svaseq
                        (dat, mod, mod0, 
                        n.sv=
                        2
                        )
                    




                        ## Number of significant surrogate variables is:  2
## Iteration (out of 5 ):1  2  3  4  5
                    




                        svseq$sv
                    




                        ##	      [,1]	  [,2]
## [1,]  0.2481108 -0.52600157
## [2,]  0.2629867 -0.58115433
## [3,]  0.1502704  0.27428267
## [4,]  0.2023800  0.38419545
## [5,] -0.6086586 -0.07854931
## [6,] -0.6101210 -0.02923693
## [7,]  0.1788509  0.25708985
## [8,]  0.1761807  0.29937417
                    


**Figure 16.**
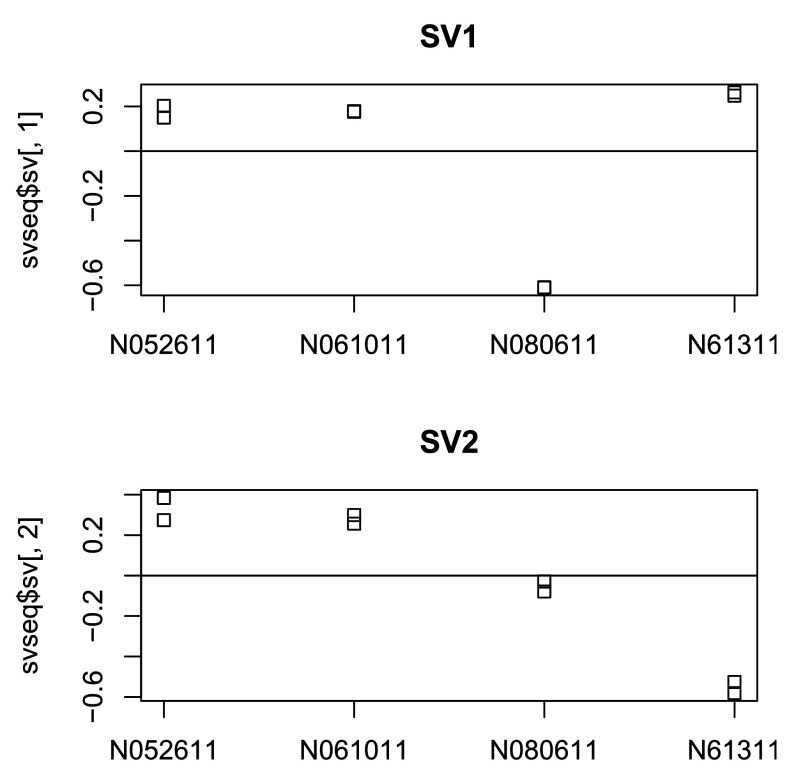
Surrogate variables 1 and 2 plotted over cell line. Here, we know the hidden source of variation (cell line), and therefore can see how the SVA procedure is able to identify sources of variation which are correlated with cell line.



Because we actually do know the cell lines, we can see how well the SVA method did at recovering these variables ( Figure 16).

                        par
                        (
                        mfrow=c
                        (
                        2
                        ,
                        1
                        ),
                        mar=c
                        (
                        3
                        ,
                        5
                        ,
                        3
                        ,
                        1
                        ))

                        stripchart
                        (svseq$sv[,
                        1
                        ] ~ dds$cell,
                        vertical=
                        TRUE
                        ,
                        main=
                        “SV1"
                        )

                        abline
                        (
                        h=
                        0
                        )

                        stripchart
                        (svseq$sv[,
                        2
                        ] ~ dds$cell,
                        vertical=
                        TRUE
                        ,
                        main=
                        “SV2"
                        )

                        abline
                        (
                        h=
                        0
                        )
                    


Finally, in order to use SVA to remove any effect on the counts from our surrogate variables, we simply add these two surrogate variables as columns to the *DESeqDataSet* and then add them to the design:



                        ddssva <- dds
ddssva$SV1 <- svseq$sv[,
                        1
                        ]
ddssva$SV2 <- svseq$sv[,
                        2
                        ]

                        design
                        (ddssva) <- 
                        ~ 
                        SV1 + SV2 + dex
                    


We could then produce results controlling for surrogate variables by running *DESeq* with the new design:



                        ddssva <- 
                        DESeq
                        (ddssva)
                    


## Time course experiments

*DESeq2* can be used to analyze time course experiments, for example to find those genes that react in a condition-specific manner over time, compared to a set of baseline samples. Here we demonstrate a basic time course analysis with the *fission* data package, that contains gene counts for an RNA-seq time course of fission yeast ^26^. The yeast were exposed to oxidative stress, and half of the samples contain a deletion of the gene *atf21*. We use a design formula that models the strain difference at time 0, the difference over time, and any strain-specific differences over time (the interaction term strain:minute).



                    library
                    (
                    "fission"
                    )

                    data
                    (
                    "fission"
                    )
ddsTC <- 
                    DESeqDataSet
                    (fission, ~ strain + minute + strain:minute)
                


The following chunk of code performs a likelihood ratio test, where we remove the strain-specific differences over time. Genes with small *p* values from this test are those which at one or more time points after time 0 showed a strain-specific effect. Note therefore that this will not give small *p* values to genes that moved up or down over time in the same way in both strains.



                    ddsTC <- 
                    DESeq
                    (ddsTC, 
                    test=
                    "LRT"
                    , 
                    reduced = ~ 
                    strain + minute)
resTC <- 
                    results
                    (ddsTC)
resTC$symbol <- 
                    mcols
                    (ddsTC)$symbol

                    head
                    (resTC[
                    order
                    (resTC$padj),],
                    4
                    )
                




                    ## log2 fold change (MLE): strainmut.minute180
## LRT p-value: '~ strain + minute + strain:minute' vs '~ strain + minute'
## DataFrame with 4 rows and 7 columns
##		 baseMean log2FoldChange      lfcSE       stat	     pvalue	    padj	symbol
##		<numeric>      <numeric>  <numeric>  <numeric>    <numeric>    <numeric>   <character>
## SPBC2F12.09c  174.6712    -2.65763737  0.7498270   99.23199 7.671942e-20 5.186233e-16         atf21
## SPAC1002.18   444.5050    -0.05118463  0.2030554   57.72116 3.590886e-11 1.213719e-07          urg3
## SPAC1002.19   336.3732    -0.39267927  0.5749887   43.26296 3.268243e-08 7.364441e-05          urg1
## SPAC1002.17c  261.7731    -1.13882844  0.6072772   39.13718 2.228530e-07 3.766216e-04          urg2
                


This is just one of the tests that can be applied to time series data. Another option would be to model the counts as a smooth function of time, and to include an interaction term of the condition with the smooth function. It is possible to build such a model using spline basis functions within R.

**Figure 17.**
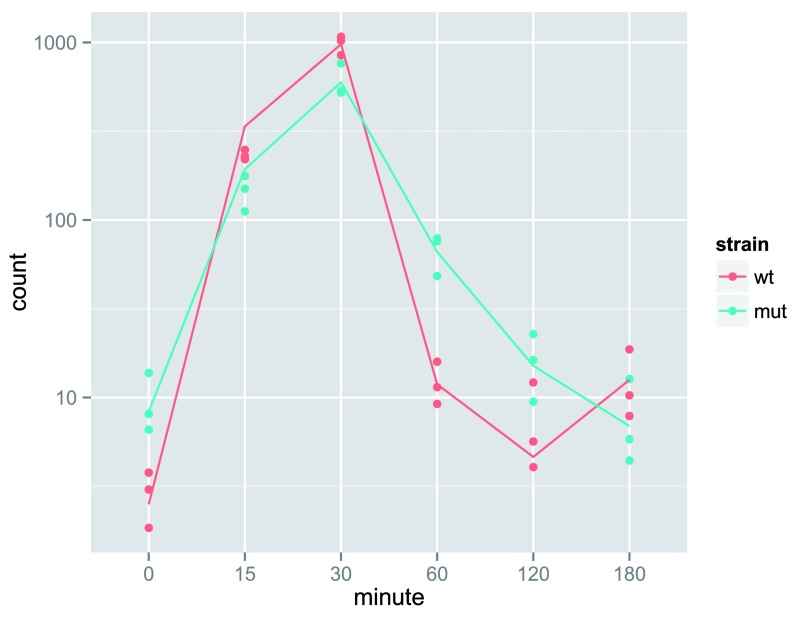
Normalized counts for a gene with condition-specific changes over time.



We can plot the counts for the groups over time using *ggplot2*, for the gene with the smallest adjusted *p* value, testing for condition-dependent time profile and accounting for differences at time 0 ( Figure 17). Keep in mind that the interaction terms are the *difference* between the two groups at a given time after accounting for the difference at time 0.

                    data <- 
                    plotCounts
                    (ddsTC, 
                    which.min
                    (resTC$padj),
		     
                    intgroup=c
                    (
                    "minute"
                    ,
                    “strain"
                    ), 
                    returnData=
                    TRUE
                    )

                    ggplot
                    (data, 
                    aes
                    (
                    x=
                    minute, 
                    y=
                    count, 
                    color=
                    strain, 
                    group=
                    strain)) +

                    geom_point
                    () + 
                    stat_smooth
                    (
                    se=
                    FALSE
                    ,
                    method=
                    “loess"
                    ) + 
                    scale_y_log10
                    ()
                


Wald tests for the log2 fold changes at individual time points can be investigated using the test argument to *results*:



                    resultsNames
                    (ddsTC)
                




                    ## [1] "Intercept"	    "strain_mut_vs_wt"	 "minute_15_vs_0"      "minute_30_vs_0"
## [5] "minute_60_vs_0"	    "minute_120_vs_0"	 "minute_180_vs_0"     "strainmut.minute15"
## [9] “strainmut.minute30" "strainmut.minute60" "strainmut.minute120" "strainmut.minute180"
                




                    res30 <- 
                    results
                    (ddsTC, 
                    name=
                    “strainmut.minute30"
                    , 
                    test=
                    “Wald"
                    )
res30[
                    which.min
                    (resTC$padj),]
                




                    ## log2 fold change (MLE): strainmut.minute30
## Wald test p-value: strainmut.minute30
## DataFrame with 1 row and 6 columns
##		 baseMean log2FoldChange     lfcSE       stat       pvalue      padj
##		<numeric>      <numeric> <numeric>  <numeric>    <numeric> <numeric>
## SPBC2F12.09c     174.6712      -2.601034 0.6314737   -4.11899 3.805364e-05 0.2572426
                


We can furthermore cluster significant genes by their profiles. We extract a matrix of the shrunken log2 fold changes using the *coef* function:



                    betas <- 
                    coef
                    (ddsTC)

                    colnames
                    (betas)
                




                    ## [1] "Intercept"	    "strain_mut_vs_wt"	 "minute_15_vs_0"      "minute_30_vs_0"
## [5] "minute_60_vs_0"	    "minute_120_vs_0"	 "minute_180_vs_0"     "strainmut.minute15"
## [9] "strainmut.minute30" "strainmut.minute60" "strainmut.minute120" "strainmut.minute180"
                


**Figure 18.**
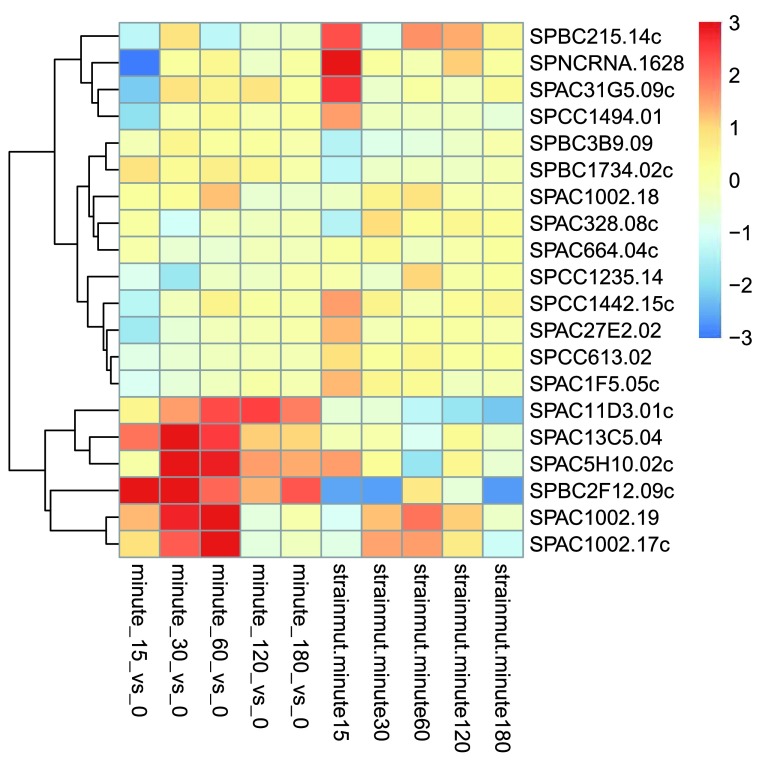
Heatmap of log2 fold changes for genes with smallest adjusted *p* value. The bottom set of genes show strong induction of expression for the baseline samples in minutes 15–60 (red boxes in the bottom left corner), but then have slight differences for the mutant strain (shown in the boxes in the bottom right corner).



We can now plot the log2 fold changes in a heatmap ( Figure 18).

                    library
                    (
                    "pheatmap"
                    )
topGenes <- 
                    head
                    (
                    order
                    (resTC$padj),
                    20
                    )
mat <- betas[topGenes, -
                    c
                    (
                    1
                    ,
                    2
                    )]
                




                    thr <- 
                    3

                    mat[mat < -thr] <- -thr
mat[mat > thr] <- thr

                    pheatmap
                    (mat, 
                    breaks=seq
                    (
                    from=
                    -thr, 
                    to=
                    thr, 
                    length=
                    101
                    ),
	  
                    cluster_col=
                    FALSE
                    )
                


## Session information

As the last part of this document, we call the function *sessionInfo*, which reports the version numbers of R and all the packages used in this session. It is good practice to always keep such a record of this as it will help to track down what has happened in case an R script ceases to work or gives different results because the functions have been changed in a newer version of one of your packages. By including it at the bottom of a script, your reports will become more reproducible.

The session information should also *always* be included in any emails to the Bioconductor support site along with all code used in the analysis.



                    sessionInfo()
                




                    ## R version 3.2.1 (2015-06-18)
## Platform: x86_64-apple-darwin13.4.0 (64-bit)
## Running under: OS X 10.10.3 (Yosemite)
##
## locale:
## [1] en_US.UTF-8/en_US.UTF-8/en_US.UTF-8/C/en_US.UTF-8/en_US.UTF-8
##
## attached base packages:
##  [1] grid	  stats4    parallel  stats     graphics  grDevices datasets  utils     methods
## [10] base
##
## other attached packages:
##  [1] fission_0.102.0		  sva_3.14.0		    mgcv_1.8-7
##  [4] nlme_3.1-122		  Gviz_1.12.1		    org.Hs.eg.db_3.1.2
##  [7] RSQLite_1.0.0		  DBI_0.3.1		    genefilter_1.50.0
## [10] ggplot2_1.0.1		  PoiClaClu_1.0.2	    RColorBrewer_1.1-2
## [13] pheatmap_1.0.7		  DESeq2_1.8.1		    RcppArmadillo_0.5.400.2.0
## [16] Rcpp_0.12.0		  BiocParallel_1.2.20	    GenomicAlignments_1.4.1
## [19] GenomicFeatures_1.20.3	  AnnotationDbi_1.30.1	    Biobase_2.28.0
## [22] Rsamtools_1.20.4	  Biostrings_2.36.4	    XVector_0.8.0
## [25] airway_0.102.0		  GenomicRanges_1.20.6	    GenomeInfoDb_1.4.2
## [28] IRanges_2.2.7		  S4Vectors_0.6.4	    BiocGenerics_0.14.0
## [31] knitr_1.11		  BiocStyle_1.6.0	    rmarkdown_0.8
##
## loaded via a namespace (and not attached):
##  [1] splines_3.2.1		  Formula_1.2-1	    	    latticeExtra_0.6-26
##  [4] BSgenome_1.36.3		  yaml_2.1.13	    	    lattice_0.20-33
##  [7] biovizBase_1.16.0	  digest_0.6.8	    	    colorspace_1.2-6
## [10] htmltools_0.2.6		  Matrix_1.2-2	    	    plyr_1.8.3
## [13] XML_3.98-1.3		  biomaRt_2.24.0	    zlibbioc_1.14.0
## [16] xtable_1.7-4		  scales_0.3.0	    	    annotate_1.46.1
## [19] nnet_7.3-10		  proto_0.3-10	    	    survival_2.38-3
## [22] magrittr_1.5		  evaluate_0.7.2	    MASS_7.3-43
## [25] foreign_0.8-66		  tools_3.2.1	    	    formatR_1.2
## [28] matrixStats_0.14.2	  stringr_1.0.0	    	    munsell_0.4.2
## [31] locfit_1.5-9.1		  cluster_2.0.3	    	    lambda.r_1.1.7
## [34] futile.logger_1.4.1	  RCurl_1.95-4.7	    dichromat_2.0-0
## [37] VariantAnnotation_1.14.13 bitops_1.0-6	    	    labeling_0.3
## [40] gtable_0.1.2		  reshape2_1.4.1	    gridExtra_2.0.0
## [43] rtracklayer_1.28.9	  Hmisc_3.16-0	    	    futile.options_1.0.0
## [46] stringi_0.5-5		  geneplotter_1.46.0	    rpart_4.1-10
## [49] acepack_1.3-3.3
                

