# Supplementary material for: RNA-Seq workflow: gene-level exploratory analysis and differential expression
Source: F1000Res. 2015 Oct 14;4:1070. [Version 1] doi: 10.12688/f1000research.7035.1 (PMC4670015; doi:10.12688/f1000research.7035.1)
Supplement: Peer review history

# Peer review: Referee response for version 1

**DOI:** 10.5256/f1000research.7573.r10806

**Published:** 2015-12-01

**Authors:** Zhijin Wu

**Affiliations:** 1 Department of Biostatistics, Brown University, Providence, RI, USA

I find the workflow a very useful document, especially in teaching someone with limited R/Bioconductor experience, starting from the count table. In addition to DE analysis, the workflow included exploratory analysis and some diagnostics -- again, very handy as teaching material.

A few comments:

1. I like the example using a subset of reads in "locating alignment files" so alignment can be done instantly in a toy example. It would be more apparent, that in addition to having very small BAM files, if it is explicitly explained that a small GTF file is created for this example. The subsection before, on "Aligning reads to a reference genome", is harder to reproduce. Unlike the examples below, there is no toy fastq file here. It would be nice to either have some toy examples here as well, or to provide the links to some actual fastq files. The link to the GEO entry is provided, which does not directly point to fastq files.
2. To make the workflow even easier to follow and reproduce, maybe a list of required packages can be provided at the very beginning.
3. I agree with Dr. Risso that at least the main steps in the DE analysis should be mentioned, so that the user understands the major components that affect the analysis. The main workflow cannot include too many examples, but possible alternatives in normalization (for example, different choices in adjusting for sequencing depth and one may consider adjusting for more than just depth) and in dispersion estimation can be simply mentioned here so that users can check these out by themselves.
4. In the airway example, there is one important detail that is worth having a checkpoint. Here,
  "Because we used a column of *sampleTable* to produce the *bamfiles* vector, we know the columns of *se* are in the same order as the rows of sampleTable. " The authors emphasized this later with the recommendation of keeping the column (sample) information in a separate file, and "making sure that the rows correspond to the columns of the *SummarizedExperiment". *
  I would recommend a checkpoint here (for example, by comparing *rownames(colData(se)*) and *rownames(sampleTable)), *so that the users will always confirm the correct order. If this is messed up, all following analysis is a waste.
5. Regrading DE and multiple testing: It is worth mentioning that the "p-value" returned is a *nominal p-value*. The Wald test p-values are not necessarily valid p-values in all experiments. Though "by the definition of the p value, we expect up to 5% of the genes to have a p value below 0.05", this only applies to valid p-values.   And if FDR is computed by adjusting nominal p-values, the *nominal FDR* may not be actual FDR either.
6. In diagnostics, Fig 14 is a useful tool. We find that the joint distribution the p-values and baseMean provides similar information --  there is no power for genes with very low base Mean. However, one would not want to guide filtering after the analysis has already been done. Thought the authors explicitly wrote that the filtering should be independent, placing the example here may leave a reader with the impression that filtering can be done post hoc. The fact that genes with low counts have low power can be established without analyzing the actual data, and we recommend making the filtering decision truly independently, for example, using simulation in the design stage ^1^.

Minor issues:

1. I wonder what the "avgLength" is in the sampleTable. SRR1039513 has avgLength 87 but if I read in the sam file I see that every read has length 63 just like the others.
2. The format of the R code is not consistent, such that some of these can be copied correctly, and some not (mostly involving quotation marks). For example in the second lines of R code,
*dir <- system.file("extdata", package=“airway", mustWork=TRUE)*, the quotation around "airway".

**

I have read this submission. I believe that I have an appropriate level of expertise to confirm that it is of an acceptable scientific standard.

## References

[1] WuHWangCWuZ: PROPER: comprehensive power evaluation for differential expression using RNA-seq. *Bioinformatics*.2015;31(2) : 1070 233-41 10.1093/bioinformatics/btu640 25273110PMC4287952

# Peer review: Referee response for version 1

**DOI:** 10.5256/f1000research.7573.r10805

**Published:** 2015-10-27

**Authors:** Davide Risso

**Affiliations:** 1 Division of Biostatistics, School of Public Health, Li Ka Shing Center, University of California, Berkeley, Berkeley, CA, USA

Love and colleagues describe a typical RNA-seq gene-level differential expression workflow, using their popular DESeq2 Bioconductor package, as well as other core and contributed Bioconductor packages. The paper is a valuable resource for researchers that are new to RNA-seq differential expression (DE) statistical analysis and/or want to learn how to carry out such analysis within R/Bioconductor.

The advantage of the presented workflow over *ad-hoc *pipelines is that (almost) the whole analysis (from aligned reads to DE results)  is carried out within the open source Bioconductor project, facilitating transparency and reproducibility. I was pleased to be able to completely reproduce all the results and figures of the paper on my machine (although I was able to download the .Rmd file only from the Bioconductor and not from the F1000Research article page -- see next paragraph).

I have only a few points that the authors should address.

1. Most of the critical statistical steps of the DE analysis are "hidden" inside a single call to the "DESeq" wrapper function. I can see why the authors created and make use of this function in the workflow, as a typical practitioner will not need to worry about the internal computations. However, the authors should consider adding a section illustrating the steps carried out by the DESeq function (normalization, dispersion estimation, model fitting), as this will have a great educational value.
2. Right below Figure 6 (mid page 18 in the pdf) the authors state that the model should account for "differences between cells [...] by using a paired design. [...] We are already set up for this design by assigning the formula ~ cell + dex earlier." The term "paired design" usually refers to a design where the same units are tested before and after a treatment, and the effect of the treatment is usually tested by modeling the differences between the measurements before and after. The specified formula describes a factorial design, where the effects of treatment and cell line are included as main effects in the model. Please make sure that the right terminology is used.
3. I found the section on independent filtering a bit confusing. Reading the fourth paragraph of the section (first paragraph of page 28 in the pdf), I understand that DESeq2 will decide how many genes to filter out by maximizing the number of genes with a low adjusted p-value. However, the next paragraph states that this type of filtering is permissible only if independent of the test statistics. How can the filtering be independent if the threshold is chosen by maximizing the number of significant genes?
4. It would be a nice addition to the manuscript to have a concluding paragraph describing how to download and reproduce the workflow.

Minor issues:

- The first time the authors mention Figure 1 they are referring to Figure 1 of a different paper, hence this should not link to the authors' Figure 1.
- Fourth paragraph of "The DESeqDataSet, sample information, and the design formula": the authors write colData(dds) although dds is not yet defined.
- The third paragraph of page 12 (of the pdf) is a bit confusing. Perhaps, it could be rephrased in terms of unsupervised problems. It's not clear why somebody with a supervised problem would want to omit the design at the EDA stage.
- Figure 4: the authors say that one should use unnormalized counts to compute the Poisson distances. Wouldn't this affect the heatmap if there are large sequencing depth differences between the samples? Or is the function internally adjusting for sequencing depth?
- The first paragraph of the "PCA plot" section should be re-written more clearly.
- The "reduced" argument of the DESeq function, used in the "Time course experiments" section, deserves a brief explanation for those readers that are not familiar with Likelihood Ratio Tests.

I have read this submission. I believe that I have an appropriate level of expertise to confirm that it is of an acceptable scientific standard.
